# Ethnographic research as an evolving method for supporting healthcare improvement skills: a scoping review

**DOI:** 10.1186/s12874-021-01466-9

**Published:** 2021-12-05

**Authors:** Georgia B. Black, Sandra van Os, Samantha Machen, Naomi J. Fulop

**Affiliations:** Department of Applied Health Research, UCL, London, UK

**Keywords:** Ethnography, Qualitative research, Healthcare improvement

## Abstract

**Background:**

The relationship between ethnography and healthcare improvement has been the subject of methodological concern. We conducted a scoping review of ethnographic literature on healthcare improvement topics, with two aims: (1) to describe current ethnographic methods and practices in healthcare improvement research and (2) to consider how these may affect habit and skill formation in the service of healthcare improvement.

**Methods:**

We used a scoping review methodology drawing on Arksey and O’Malley’s methods and more recent guidance. We systematically searched electronic databases including Medline, PsychINFO, EMBASE and CINAHL for papers published between April 2013 – April 2018, with an update in September 2019. Information about study aims, methodology and recommendations for improvement were extracted. We used a theoretical framework outlining the habits and skills required for healthcare improvement to consider how ethnographic research may foster improvement skills.

**Results:**

We included 274 studies covering a wide range of healthcare topics and methods. Ethnography was commonly used for healthcare improvement research about vulnerable populations, e.g. elderly, psychiatry. Focussed ethnography was a prominent method, using a rapid feedback loop into improvement through focus and insider status. Ethnographic approaches such as the use of theory and focus on every day practices can foster improvement skills and habits such as creativity, learning and systems thinking.

**Conclusions:**

We have identified that a variety of ethnographic approaches can be relevant to improvement. The skills and habits we identified may help ethnographers reflect on their approaches in planning healthcare improvement studies and guide peer-review in this field. An important area of future research will be to understand how ethnographic findings are received by decision-makers.

**Supplementary Information:**

The online version contains supplementary material available at 10.1186/s12874-021-01466-9.

## Background

Research can help to support the practice of healthcare improvement, and identify ways to “improve improvement” [1]. Ethnography has been identified particularly as a research method that can show what happens routinely in healthcare, and reveal the ‘*what* and *how* of improving patient care [2]. Ethnography is not one method, but a paradigm of mainly qualitative research involving direct observations of people and places, producing a written account of natural or everyday behaviours and ideas [3]. Ethnographic research can identify contextual barriers to healthcare improvement. For example, Waring and colleagues suggested that hospital discharge could be improved by allowing staff to have more opportunities for informal communication [4].

There have been advances in ethnographic methods that support its role in supporting healthcare improvement. Multi-site, collaborative modalities of ethnography have evolved that suit the networked nature of modern healthcare [5]. Similarly, rapid ethnographic approaches (e.g. Bentley et al. [6];) meet the needs of improvement activities to produce findings within short timeframes [7]. However, the production of sustained ethnographic fieldwork has waned in response to demands for rapid evidence [6, 8, 9]. Critics of rapid ethnographic methods worry that they are diluting ethnography within applied contexts more widely [5, 10].

The relationship between ethnography and healthcare improvement has been the subject of methodological concern [8]. The first concern is that some research identified as *ethnography* does not fit within the ethnographic paradigm, merely collecting observational data without a theoretical analysis, interpretation or researcher reflexivity [11]. A second concern is whether the topics of ethnographic inquiry produce findings that are seen as useful for improvement [12], particularly if they do not make explicit recommendations or produce checklists [8, 13–15]. Authors fear that ethnographic findings that capture complexity [16] and expose taken-for-granted behaviours and phenomena [14, 17] may be too abstract to be relevant to healthcare improvement [8]. However, these critiques position ethnographic research as a product which may be taken up by healthcare improvers, rather than seeing ethnographic work itself as an improvement activity. We take the view that healthcare improvement aims to change human behaviour to improve patient care, and is therefore reliant on the development of particular skills and habits (such as good communication) [18]. We would consider that engaging in ethnographic research may support skill development and habit formation that serves healthcare improvement.

In the literature of ethnography in healthcare improvement, there is not much discussion of the close relationship between methodological features of ethnographic research, and their impact on improvement skills. The aim of this paper is twofold: (1) to describe current ethnographic methods and practices in healthcare improvement research and (2) to consider how these may affect habit and skill formation in the service of healthcare improvement [19].

## Methods

This is a scoping review following the methods outlined by Arksey & O’Malley and later refined by Levac et al., [20, 21] including a systematically conducted literature review and reported in accordance with the Preferred Reporting Items for Systematic reviews and Meta-Analyses extension for Scoping Reviews (PRISMA-ScR; see Additional file 1 for PRISMA checklist). No protocol was published for this review. Our literature search and analyses were conducted iteratively, searching reference lists and undertaking discussions with colleagues about key lines of argument. We also held a workshop at Health Services Research UK conference in 2018 on this topic to gain a wide range of stakeholder views.

### Systematic retrieval of empirical papers and purposive sampling

Our search strategy was designed to capture a wide range of approaches to ethnography from different journals, healthcare settings and types of research environment. It was not our aim to capture every study using this methodology, but to map the current field. Thus we did not search grey literature, books or monographs. The search strategy was developed and piloted in consultation with a health librarian. Medline (on OVID platform), PsychINFO, CINAHL and EMBASE databases were searched, and six journals were hand-searched, including: BMJ Quality & Safety, Social Science and Medicine, Medical Anthropology, Cochrane library, Sociology of Health and Illness and Implementation Science. These databases were searched between dates April 2013 – April 2018 and an update was performed in September 2019 using the search terms outlined in Additional file 2. We limited the search to these dates in order to capture the most recent methodological characteristics of ethnographic studies in this field.

We screened titles and then abstracts according to the inclusion and exclusion criteria detailed in Table 1. We included studies which self-identified as using *ethnography* or *ethnographic methods* rather than using our own criteria. This is because ethnography can be hard to define, and use of criteria may risk excluding papers which exemplify the sorts of tensions and workarounds we are trying to capture.

**Table 1:** Inclusion and exclusion criteria

	Inclusion criteria	Exclusion criteria
Method	• Stated to be using ethnographic methods of any kind	• Meta-ethnography or meta-synthesis • Scoping review or other review methodologies • Interviewing or observational work alone without reference to ethnographic lens
Subject matter	• Studies relating to healthcare topics or from an applied healthcare discipline, as defined by the specific search terms	• Public health topics (health promotion, screening, vaccination, communicable disease management, etc.) • Health-related topics that are not within health service context, such as o self-management techniques, care homes, social care, peer support groups, refugee centres, day care, community interventions, prisons o health beliefs, cultural attitudes, patient views, disease experiences o trial acceptability, research acceptability o ethnography related to basic science • Social care • Organisational studies that are not situated in health service settings • Studies about ethnographic methodology with no specific reference to health or healthcare
Study design	• Peer-reviewed publications • Studies that state their use of ethnographic methods	• Commentary, letter, response, critical review • Book review

**Fig. 1:**
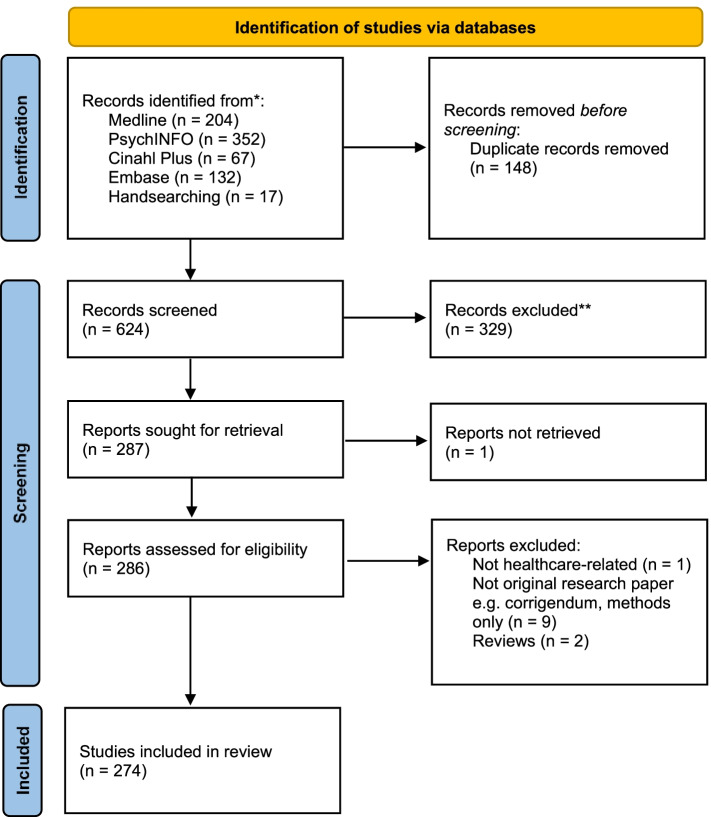
PRISMA statement of all references retrieved, screened and included in the scoping review

The retrieved papers were screened by GB, SVO and SM based on inclusion and exclusion criteria (Table 1). The total number of papers after screening titles, abstracts and full texts was 274 (Fig. 1).

### Numerical charting

Characteristics of each paper, such as title, authors, journal, year, country and healthcare subject area were extracted (see Table 2).

**Table 2:** Characteristics of studies in review

Method summary	
Focused ethnography	25
Thematic analysis	21
Grounded theory study	15
Case study	13
Mixed methods	13
Institutional ethnography	12
Critical ethnography	12
Content analysis	8
Constant comparison	7
Discourse analysis	6
Auto-ethnography	2
Other	107
Region^a^	
Middle East	5
South America	11
Asia	15
Africa	22
Australasia	33
Europe (excl. UK)	47
UK	74
North America	95
Healthcare subject area	
Clinical communication	3
HIV-AIDS	3
Intensive Care Unit	7
Medication prescribing and management	8
Cancer	10
Paediatrics	10
Surgery and orthopaedics	10
Patient safety	11
Emergency medicine and acute care	12
Chronic illness	12
Family doctors, primary care and general practice	12
Nursing practice	13
Healthcare technology	14
Maternity care and reproductive medicine	15
Quality of care improvement and healthcare reform	18
Mental health and psychiatry	19
Dementia, care of the elderly, end of life care, palliative care	20
No info/other	86

^a^some studies have been allocated to more than one region

### Thematic analysis and development

We coded all 274 papers using NVivo software for stated aims and recommendations. This included close reading, and retrieval of key ideas and quotations from the papers that exemplified key ideas in relation to healthcare improvement, methodology and the authors’ reflections on these. The coded extracts of aims and recommendation in conjunction with the closer reading of the sub-sample were used to inductively develop conceptual ideas, such as how the corpus of papers explicitly aimed to contribute to healthcare improvement, and if not, how this affected the types of conclusions drawn. Some papers were read in greater depth to understand how the authors’ methods related to their findings and conclusions. In order to consider how ethnography supports habits and skills associated with healthcare improvement, we drew on a framework which identifies five habits of ‘improvers’: creativity, learning, systems thinking, resilience and influencing [19]. Applying this model to our selected papers, we mapped traits or approaches to the ethnographic studies that exemplified these habits either in the authors, or as part of developing these habits in others (e.g. healthcare decision-makers and professionals). Thematic interpretations and lines of argument were generated and discussed by all the authors.

## Results

### Overview of study characteristics

The included studies covered a wide range of ethnographic methodologies and healthcare subjects, published internationally (Table 2) in predominantly social science and clinical journals (see Additional file 3). The full list of the 274 included studies is available in Additional file 4.

Most studies described themselves as an ‘ethnography’ or ‘ethnographic’, although some described their methodology as ‘mixed methods’ including ethnographic components. For example, Collet et al. conducted a mixed methods participatory action research study using observations to produce an “ethnographic description” [22].

Almost all studies relied on observation and interviews as the main data sources. It was not always specified whether researchers took a participant or non-participant approach to observation. There were some examples of other data sources e.g. video data, surveys, documents, field notes, diaries, and artefacts. A few examples contained a paucity of data, such as only video data [23], limited fieldwork [24], a small number of interviewees [25], or reliance on focus group data alone [26]. Methods associated with qualitative methodology (but not necessarily ethnographic) were also used, such as data ‘saturation’ to denote that additional data did not provide new insights into the topic [27].

There were a number of minority or unusual ethnographic variations:

- Quantitative ethnography [23]: temporal coding of physicians' workflow and interaction with the electronic health record system, and their patient.
- Cognitive ethnography [28]: *“identifying and elaborating distributed cognitive processes that occur when an individual enacts purposeful improvements in a clinical context”.*
- Street-level organizational ethnography [29]: intensive case study methods to explore the implications of healthcare policy at a street level.
- Phenomenological ethnographies [30]: focussing on the lived experience and meanings associated with a phenomenon.
- Geo-mapping [31]: geomapping of selected service data to define Latino immigrant community before conducting interviews and observations.

### Use of different types of ethnography to support healthcare improvement

We found that many studies used methods that could identify issues relating to power and vulnerability, with potential relevance to how healthcare improvement problems are defined and solved, and by whom [1]. For example we noted a significant minority of studies using institutional and critical ethnography, mostly in vulnerable populations (see Table 3). These studies were explicitly attentive to systems and power relations, rather than on individual practices. We suggest that the use of geographically-oriented methods such as geo-mapping and street-level organisational ethnography are also attentive to the power structures inherent in place and space, and could be relevant to other geographical healthcare improvement topics such as networked healthcare systems, care at home and patient travel for treatment.

**Table 3:** Ethnographic methodology and its relevance to healthcare improvement

Ethnographic methodology used	Description	Example paper	Relevance to healthcare improvement
Video-reflexive ethnographic study	Collecting in-depth data on intimate or micro-interactions	Patients’ and families’ perspectives of patient safety at the end of life: a video-reflexive ethnography study. (Collier, Sorensen, Iedema, 2016) [32]	• Able to capture complexity in delivery of healthcare. • Irrefutable basis for improving healthcare delivery from the 'bottom up' • Video footage played back to participants. • Video footage challenges the taken for granted aspects of practice individuals may not be aware of
Peer ethnography	Peers collecting data from excluded or vulnerable populations	Using Peer Ethnography to address health disparities among young Black and Latino men who have sex with men. (Mutchler et al., 2013) [33]	• Improves access to marginalised groups • Data collection on healthcare topics that may only happen between peers (for example, discussions about substance use with men who have sex with men)
Focussed ethnography	Focus on a discrete community or organisation or social phenomena; problem-driven	Culture of Care for Infants with Neonatal Abstinence Syndrome: A Focused Ethnography. (Nelson, 2016) [34]	• Method often used in nursing research • Intense, short-term observation and interview data collection provides rich and thick description of culture of care • Rapid feedback loop into improvement through focus and insider status
Critical ethnography	Projects with vulnerable populations and/or political improvement agendas	Nursing casualization and communication: a critical ethnography. (Batch and Windsor, 2014) [35]	• Method gives focus to power, communicative distortions and context • 'Critical' element turned the focus to structures and situations of power and dominance that underpinned nursing culture
Institutional ethnography	Research studying complex social issues and projects that aim to achieve meaningful social change at the nexus of health professions education and other social systems	Homelessness, health, and literacy: an institutional ethnographic study of the social organization of health care in Ontario, Canada. (Hughes, 2018) [36]	• Insights to explicate the complex and invisible relations that exist being people, place, and things. • Powerful tool to explore the multi-layer entity of health care
Qualitative methodology incorporated into ethnographic studies			
Grounded theory	Researcher co-constructs theories with the research participants, building the theory de novo from iterative data collection	Using an emic and etic ethnographic technique in a grounded theory study of information use by practice nurses in New Zealand. (Hoare et al., 2013) [37]	• Focus on theory generation supports generalisability of healthcare improvement recommendations • Incorporating of grounded theory techniques such as memoing heightens reflexivity [38] • Gives priority to the studied phenomena rather than the study setting
Thematic analysis	Flexible qualitative analysis method of deriving themes from data through systematic coding procedures	Taking the heat or taking the temperature? A qualitative study of a large-scale exercise in seeking to measure for improvement, not blame. (Armstrong et al., 2018) [39]	• Findings are (potentially) accessible to different audiences due to thematic presentation • Allows analysis of observation and interview data from a diverse sample of organisations • Can thematically explore people's views as well as see what they did in practice

The high prevalence of ethnographic studies with vulnerable populations (e.g. psychiatry, end of life care) suggests that ethnography is also being conceptualised as an emancipatory method, reversing healthcare power structures in its focus. This has been a traditional focus of ethnography since social changes in power and representation in the 1970s, incorporated into the development of healthcare research methodology [40, 41]. Some methods used were calculated to maximise the potential for supporting vulnerable groups, for example, Nightingale et al. [42] used focused ethnography (prolonged fieldwork in a small number of settings) to look at patient-professional interactions in paediatric chronic illness settings. The authors suggested that focussed ethnography is particularly suited to settings where fostering trust is essential. We would also suggest that ethnography may be particularly suited to settings in which participants are less able to verbalise their experiences.

The reviewed studies suggested that video ethnography can support healthcare improvement at a team level. For example, Stevens et al. [43] promoted video ethnography as a way to capture in-depth data on intimate interactions, in their study of elective caesareans. The video data allowed them to make use of timing data (e.g. of certain actions), physical positioning of different actors and equipment, and verbatim dialogue recording. The video data also suited the technical nature of the procedure, which was relatively time-limited. This form of data collection may not suit environments where healthcare activities are more spread out.

#### The impact of healthcare practitioner involvement in ethnographic fieldwork and findings

We noted that the use of ethnography for healthcare improvement has led to healthcare practitioners’ widespread involvement in data collection or analysis. We suggest that this is a form of negotiation across the healthcare-academia boundary, translating from ‘real world’ to data and back again. This has potential to create rich and relevant ethnographic studies that are geared towards improvement. However, some studies were undermined by a lack of reflexivity about the dual practitioner-ethnographer role.

A significant number of papers involved healthcare practitioners in fieldwork (e.g. Abdulrehman, 2017, Hoare et al. 2013; [37, 44]). For example in Hoare et al. the lead researcher was a nurse, and wrote that they hoped *“to bring both an emic and etic perspective to the data collection by bracketing my emic sense of self as a nurse practitioner in order to become a participant observer within my own general practice*” [37]. In this study, the findings fed directly into local service improvement as the lead researcher felt compelled to *“share new ‘best practice’ information and join in the conversation.”* There was little discussion about how this affected the generalisability of the findings, and whether their recommendations were adopted.

Similarly, Bergenholz et al. [45] conducted a study where a nursing researcher completed the main fieldwork and *“assisted the nurses with practical care*.” They acknowledged that *“This may have caused limitations with regards to ‘blind spots’ in the nursing practice, but that it also gave access to a field that might be difficult for ‘outside-outsiders’ to gain*.” However, there was no commentary on where the blind spots or extra access occurred, and how this may have affected the relevance and dissemination of their findings.

### How might ethnography support healthcare improvement habits?

In this section, we evaluate the studies included in the review in terms of how their methods relate to improvement. We draw on the idea that successful improvement is based on a set of habits and their related skills acquired through experience and practice [19]. This section is structured around Lucas’s five habits of ‘improvers’: creativity, learning, systems thinking, resilience and influencing [19]. Under those headings, we describe the mechanisms by which ethnographic studies can support healthcare improvement habits, using illustrative examples.

### Resilience

Resilience is defined as being adaptable, particularly tolerating calculated risks and uncertainty, and proceeding with optimism. Being able to recover from adverse events is core to improvement, reframing them as opportunities. Adaptation and the ability to bounce back from adverse events and variation are core to improvement.

#### Tolerating the uncertainty of ethnographic data collection

While we did not relate these traits to any particular ethnographic approach in our studies, we would consider that undertaking any ethnographic project requires resilience, as data collection is inherently exploratory and uncertain. For example, Belanger et al. wanted to know how health care providers and their patients approach patient participation in palliative care decisions. The authors explicitly eschewed the pull to create guidelines or other formalised knowledge, but aimed to explore the *“unforeseen and somewhat unavoidable ways in which discursive practices prompt or impede patient participation during these interactions.”* [46]

### Creativity

Creativity is defined as working together to encourage fresh thinking by generating ideas and thinking critically.

#### Using a theoretical lens

Researchers may consider healthcare through a particular theory or framework (e.g. private ordering [47], masculine discourse [48], compassion [49]). The restriction of the theoretical lens enables critical thinking, and keeps the ethnographer creatively engaged. For example, Mylopoulos & Farhat [28] used the concept of adaptive expertise in a cognitive ethnography to explore *“the phenomenon of purposeful improvement”* in a teaching hospital. This theoretical lens revealed that clinicians were engaging in *“invisible”* improvement in their daily work, in *“specific activities such as scheduling, establishing patient relationships, designing physical space and building supporting resources”.* The authors suggested that these practices were devalued in comparison to more formal improvement activities, justifying the utility of the ‘adaptive expertise’ theory in bringing the daily improvement practices to light.

#### Challenging current problems and perspectives

We identified studies that challenged or reframed existing improvement problems e.g. Mishra [50]. This role removes the ‘blinkers’ of improvement research [51], and can ‘dissolve’ previously intractable implementation problems. For example, Boonan et al. [52] studied the practice of bar-coded medication from the perspective of nurses using the intervention. In their discussion, the authors challenge the assumption that if you introduce technology, then you will mitigate human factor risks. They highlighted that external pressures on hospitals perpetuate this perspective, and that *“nurses and patients are consequently drawn into this discourse and institutional ruling, to which they are not oblivious”.* Their recommendation was to understand the skills of nurses in tailoring technology to meet individual patients’ needs rather than trusting in systems blindly.

### Learning

Learning is defined as harnessing curiosity and using reflective processes to extract meaning from experience.

#### Inviting reflection

We noted that some studies did not make explicit recommendations for improvement, but wrote their findings in a manner that would invite reflection on its subject matter. For example, Thomas & Latimer [53] wrote that they view their role as provocateurs of new ideas, stating that their intention *“is not to propose specific policies or discourses designed to change or improve practice. More modestly, we hope that by analysing the everyday and by theorising the mundane, this article will ignite reflexive, ethical and pluralistic dialogues – and so better communication between practitioners, parents and the wider lay public – around reproductive technologies and medical conditions”* (authors’ underline; p.951-2) [53]. Others such as Mackintosh et al [54] used their discussion section to examine their results in the context of other theories and provide illumination: *“Our focus on trajectories illuminates the physiological process of birth and the unfolding pathology of illness (and death). This frame provides a means for us to link the agency of those involved in organising the care of acutely ill patients with the wider socio-political factors beyond the clinic, such as governmentality and risk (Heyman 2010, Waring 2007), death brokering (Timmermans 2005) and the medicalisation of birth and death (De Vries 1981).”* (p.264). These two examples show that ethnographic work can be offered as an opportunity for learning and reflection, without a translation to specific recommendations.

#### Supporting a more ethical, expansive, inclusive, and participatory mode of healthcare

Problem-finding is highlighted as an important part of learning in improvement [19]. Several studies paid attention to multivocality and power, using this to find problematic, unethical and exclusive practices in healthcare. For example, some studies reported previously unheard viewpoints [55–57], or identified restrictive organisational barriers and normative assumptions [58, 59]. Others promoted ethnography as a way of exploring ethics and morality [47, 60, 61], such as criticising research that prioritizes the needs of individuals over the good of society [62]. Ross et al. [63] suggested that it is also more ethical to use critical ethnography than other evaluative methods in researching vulnerable populations (e.g. neurological illness), by being able to *“explore perceived political and emancipatory implications,* [clarify] *existing power differentials and* [maintain] *an explicit focus on action”*.

Some studies directly researched power within the healthcare setting. For example, Batch and Windsor’s study of nursing workforce suggested that senior nurse leaders should use their positions to advocate for better working conditions [35], “*Manageable nurse/patient ratios, flexible patient-centred work models, equal opportunity for advancement, skill development for all and unit teamwork promotion”.* Challenging traditional cultural assumptions that have produced and reproduced stereotypes is problematic because they most often are, by their very nature, invisible. In a more critical approach, Gesbeck’s thesis [62] on diabetes care work challenges the very mechanism of achieving healthcare improvement through research, stating that *“we need to change the social and political context in which health care policy is made. This requires social change that prioritizes the good of the society over the good of the individual—a position directly opposed to the current system oriented toward profit and steeped in the ideology of personal responsibility.”*

### Systems thinking

Systems thinking is defined as seeing whole systems as well as their parts and recognising complex relationships, connections and interdependencies.

#### Suggesting reorientation to new ‘problem’ areas

We found that many ethnographic studies emphasised skills of synthesis and connection-making, reorienting improvement to different areas, for example in overarching policy recommendations (e.g. Hughes [36]; Liu et al. [64], Matinga et al. [65]), or resetting priorities. For example, Manias’ [66] ethnography of communication relating to family members' involvement in medication management in hospital suggests that *“greater attention should be played on health professionals initiating communication in proactive ways*” [p.865]. In another example, Cable-Williams & Wilson’s (2017) focussed ethnography captures cultural factors within long-term care facilities. Their discussion suggests that acknowledgement of death is under-represented in front-line practice and government policy, reorienting discussions towards an integration of living and dying care.

#### Exposing hidden practices within the everyday

We found that several studies drew attention to ‘hidden’ practices in healthcare work, allowing them to evaluated and improved. For example, we found reference to practices such as coordinating [67], repair [68], caretaking [69], scaffolding [68], tinkering [52] and bricolage [58]. We also found that some studies had new interpretations of ‘the everyday’ or ‘taken-for-granted’ (e.g. nursing culture [34, 35, 45, 70], interprofessional practice [67, 71–75]). Authors’ outputs included frameworks [76] or models [69, 71, 77, 78] that map these types of practices in a way that is helpful for intervention development or quality improvement. For example, Mackintosh et al. [54] looked at rescue practices in medical wards and maternity care settings using Strauss’s concept of the patient trajectory. Their findings highlighted the risks inherent in the wider social practices of hospital care, and suggested that improvement was needed at a level *“beyond individual and team processes and technical safety solutions.”*

### Influencing

Influencing is defined as engaging others and gaining buy-in using a range of facilitative processes.

#### Direct translation of findings to targets for improvement

Lucas suggests that to be influential, ethnographic studies need to have some empathy with clinical reality, whilst being facilitative and comfortable with conflict [19]. This was shown in ethnographic studies that made pragmatic recommendations, such as in Jensen’s study of clinical simulation. They advised that simulation might be useful in staging *“adverse event scenarios with a view to creating more controlled and safer environments.”*( 80). In MacKichan et al. [79] observations and interviews were used to understand how primary care access influenced decisions to seek help at the emergency department. The authors made empathic, actionable recommendations such as “*simplifying appointments systems and communicating mechanisms to patients.”* (p.10).

#### Evaluating the context of healthcare improvement

By capturing contextual and social aspects of healthcare improvement, ethnographic evaluations can support leaders and managers who are trying to implement improvement activities. This is a particularly helpful trait in ethnographic studies that pay attention to politics, governance and social theory in their evaluation of new interventions, “zooming out” [80] beyond the patient-clinician interaction to broader social networks. For example, Tietbohl et al. [81] investigated the difficulties of implementing a patient decision support intervention (DESI) in primary care through the theoretical lens of relational coordination between *“physician and clinical staff groups (healthcare professionals)”.* The authors’ recommended attention to the *“underlying barriers such as the relational dynamics in a medical clinic or healthcare organization”* when creating policies and programs that support shared decision-making using support interventions. This sort of insight can make it more likely that new policies or interventions will succeed. This skill was particularly fertile in the tradition of techno-anthropology, exploring technology-induced errors and the real-world interaction between people and technology, e.g. decision-support tools [81–86], the introduction of robot caregivers [87] and clinical simulations [88]. Other approaches included an investigation of one intervention or change but with a theoretical lens of inquiry.

## Discussion

### Summary of findings

This scoping review has identified the methodological characteristics of 5 years of published papers that self-identify as ethnography or ethnographic in the field of healthcare improvement. Ethnography is currently a popular research method in a wide range of healthcare topics, particularly in psychiatry, e.g. mental health, dementia and experiential concerns such as quality of life. Focused ethnography is a significant sub-group in healthcare, suggesting that messages about the importance of research timeliness have taken hold [89].

We have identified ethnographic methods reported in these papers, and considered their utility in developing skills and habits that support healthcare improvement. Specific practices associated with the ethnographic paradigm can encourage *good* habits (resilience, creativity, learning, systems thinking and influencing) in healthcare, which can support improvement. For example, using relevant theories to look at every day work in healthcare can foster creativity. The use of critical and institutional ethnography could increase skills in ‘systems thinking’ by critically evaluating how healthcare improvement problems are defined and solved, and by whom.

### Comparison with previous literature

This scoping review is the first to consider how current ethnographic methods and practices may relate to healthcare improvement. Within the paradigm of applied healthcare research, there is normative value in being ‘useful’ or ‘impactful’ in our research, which affects our prospects for funding and career success [12]. However, our review has uncovered a multitude of ways that an ethnographic study can be useful in relation to healthcare improvement, without creating actionable findings. We found a spectrum of interactions with healthcare improvement: some authors explicitly eschewed recommendations or clinical implications; others made imperative statements about required changes to policy or practice. However, this diversity was not necessarily a reflection on how ‘traditional’ the ethnographic methodology was. This challenges the paper by Leslie et al. which puts ethnographic studies in two output categories with respect to healthcare improvement: critique versus feedback [8]. Instead, we uncovered a variety of ways that ethnography can support healthcare improvement habits, such as encouraging reflection, problem-finding and exposing hidden practices in healthcare.

We did find that supporting healthcare improvement through ethnographic research can require strategic effort, however. For example, we noted that several authors wrote multiple articles based on the same project, often for different types of journal to reach different audiences such as diverse readerships in health services and academic settings. For example, Collier and colleagues published two papers based on a video ethnography of end-of-life care (both in 2016), one in a healthcare quality journal [32] and one in a qualitative research journal [76]. The former is shorter, with explicit recommendations for patient safety, whereas the latter is longer, has more detailed results and long sections on reflexivity. Similarly, Grant published an article in a sociology journal [90] and a healthcare improvement paper [91] on the same work about medication safety. The sociological paper covered *“spatio-temporal elements of articulation work”* whereas the other put forward *“key stages”* and risks, suggesting that it was more closely oriented to improvement.

There have been some considerable debates about changes in ethnographic methods and tools, with concerns about lost researcher identity, dilution of the method, and challenges to “upholding ethnographic integrity” [92] . We contest this, suggesting that new variants such as focussed and cognitive ethnography are evolving in response to the complexity of hospitals and healthcare [93], while also being highly regulated, standardised and ordered by biomedicine. Such complex environments cannot be studied and improved under one paradigm alone. Ethnographic identity and method have also been affected by the cross-pollination of ethnography with other social science paradigms and applied environments (e.g. clinical trials, technology development). Debates about theoretical and methodological choices are not only made merely with respect to healthcare improvement, but also in response to professional pressures (e.g. university requirements for impact) [12], and the mores of taste situated within the overlapping communities of practice that evaluate ethnographic healthcare research [94]. That said, we echo previous authors’ calls for attention to reflexivity, particularly in embedded or clinician-as-researcher roles [95].

Our scoping review challenges a previously expressed concern that ethnographic studies may not produce findings that are useful for improvement [10, 12, 16]. By considering different ethnographic designs in relation to skills and habits needed for improvement, we have shown that studies need not necessarily produce ‘actionable findings’ in order to make a valuable contribution. Instead, we would characterise ethnography’s role in the canon of healthcare research methodologies as a way of enhancing improvement habits such as comfort with conflict, problem-finding and connection-making.

### Strengths and limitations

This review has a number of limitations. The search may not have found all relevant studies, however the retrieved papers are intended as an exemplar rather than an exhaustive or aggregative review. The review is also limited to journal articles as evidence of researchers’ approach to improvement. This ignores many other ‘offline’ and ‘online’ activities such as meetings, presentations, blogs, books, and websites, which are conducted to disseminate findings and ideas. Our reliance on self-report for the identification of ethnographic studies will have excluded some studies within an ethnographic paradigm who chose different terms for their methodology (e.g. critical inquiry, case study). The strengths of this paper are its comprehensive coverage, incorporating all representative studies in healthcare research published within a five year period, and a wide range of ethnographic sub-types and healthcare subjects, drawn from an international pool of research communities.

## Conclusions

We did not prescribe the right way for ethnographers to engage in healthcare improvement, indeed, we have identified that a variety of approaches can be relevant to improvement. The habits we identified may help ethnographers reflect on their approaches in planning healthcare improvement studies and guide peer-review in this field. Issues of taste, traditionalism and researcher identity need to be scrutinised in favour of value and audience. An important area of future research will be to understand how ethnographic findings are received by decision-makers, and further focused reviews on the relationship(s) between ethnographic methods, quality improvement skills and improvement outcomes.

## Supplementary Information

**Additional file 1.**

**Additional file 2.**

**Additional file 3.**

**Additional file 4.**

## Data Availability

All papers included in the review are listed in Additional file 4 and are publicly available from their publishers’ websites.
