# Correction to: Ethnographic research as an evolving method for supporting healthcare improvement skills: a scoping review

**DOI:** 10.1186/s12874-022-01587-9

**Published:** 2022-04-11

**Authors:** Georgia B. Black, Sandra van Os, Samantha Machen, Naomi J. Fulop

**Affiliations:** Department of Applied Health Research, UCL, London, UK

**Correction to: BMC Med Res Methodol 21, 274 (2021)**

**https://doi.org/10.1186/s12874-021-01466-9**

Following publication of the original article [1], the authors noticed that Fig. 1 was incorrect and it needs to be updated. Presented here is the correct Figure 1. Also, the authors would like to change the number of included papers from "283" to "274" throughout the review. The original article has been updated.

**Fig. 1:**
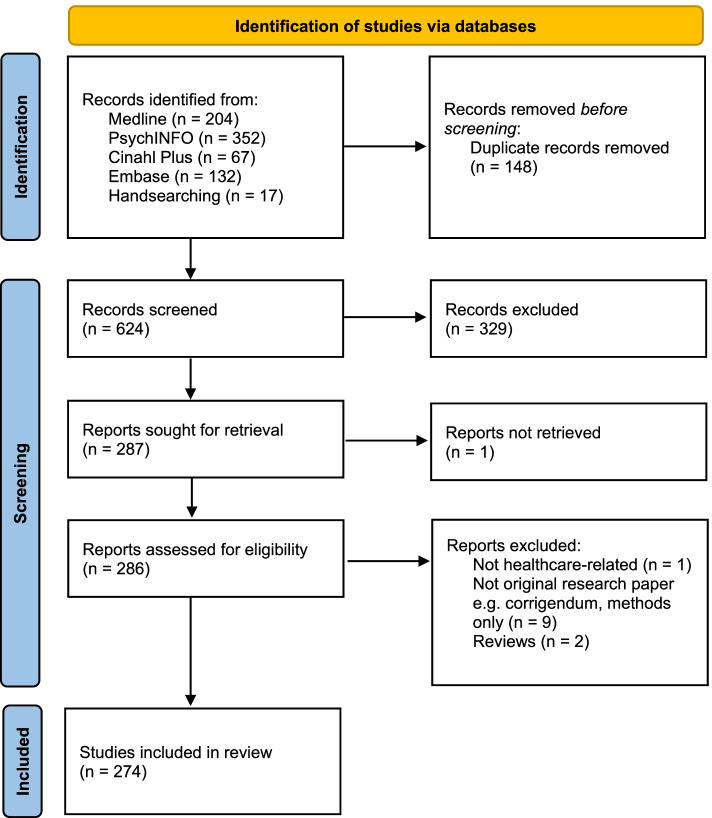
PRISMA statement of all references retrieved, screened and included in the scoping review
