# Supplementary material for: Ethnographic research as an evolving method for supporting healthcare improvement skills: a scoping review
Source: BMC Med Res Methodol. 2021 Dec 5;21:274. doi: 10.1186/s12874-021-01466-9 (PMC8647364; doi:10.1186/s12874-021-01466-9)
Supplement: Supplementary file 2 — Additional file 2. [file 12874_2021_1466_MOESM2_ESM.docx]

**Additional File 2: Search strategy**

| Resource | Search terms used | Papers retrieved |
| --- | --- | --- |
| Medline | Anthropology, Cultural/ OR ethnograph$ (in title)  AND  Total Quality Management/ or Quality Assurance, Health Care/ or Patient Safety/ or Quality Improvement/ or Safety Management/ or "Quality of Health Care"/ or Quality Indicators, Health Care/ or Medical Errors/ or Program Evaluation/ or Health Services Research/ or DIAGNOSTIC ERRORS/ or MEDICATION ERRORS/ or "Attitude of Health Personnel"/ or Organizational Culture/ or "Delivery of Health Care"/  English language, 2013-current | 204 |
| PsychINFO | exp ETHNOGRAPHY/ or exp ANTHROPOLOGY/ or "ethnograph*".m_titl.  AND  exp QUALITY CONTROL/ or exp "QUALITY OF SERVICES"/ or exp "QUALITY OF CARE"/ or exp "QUALITY OF WORK LIFE"/ or exp "Quality of Care"/ or exp Health Care Delivery/ or exp Health Service Needs/ or exp Health Care Services/ or patient safety/ or exp Decision Making/ or exp HEALTH CARE DELIVERY/ or exp Health Care Services/ or exp Hospitals/ or exp Drug Administration Methods/ or exp Intervention/ or exp Health Personnel Attitudes/ or exp Evidence Based Practice/ or exp Organizational Development/ or exp Organizational Behavior/ or exp Management/ or exp Integrated Services/ or exp Organizational Change/ or exp Organizational Effectiveness/  English language, 2013 – current | 378 |
| CINAHL Plus | Anthropology, Cultural/ OR ethnograph$ (in title)  AND  Total Quality Management/ or Quality Assurance, Health Care/ or Patient Safety/ or Quality Improvement/ or Safety Management/ or "Quality of Health Care"/ or Quality Indicators, Health Care/ or Medical Errors/ or Program Evaluation/ or Health Services Research/ or DIAGNOSTIC ERRORS/ or MEDICATION ERRORS/ or "Attitude of Health Personnel"/ or Organizational Culture/ or "Delivery of Health Care"/  English language, 2013-current  2008 – April 2018 = 67 (44 after removing duplicates after deduping against previous search) | 67 |
| Embase | Anthropology, Cultural/ OR ethnograph$ (in title)  AND  Total Quality Management/ or Quality Assurance, Health Care/ or Patient Safety/ or Quality Improvement/ or Safety Management/ or "Quality of Health Care"/ or Quality Indicators, Health Care/ or Medical Errors/ or Program Evaluation/ or Health Services Research/ or DIAGNOSTIC ERRORS/ or MEDICATION ERRORS/ or "Attitude of Health Personnel"/ or Organizational Culture/ or "Delivery of Health Care"/  English language, 2013-current  2008 – April 2018 = 67 (44 after removing duplicates after deduping against previous search) | 132 |
| Hand searching journals | ((ethnograph*) AND ("healthcare improvement" or "quality improvement" or "health care improvement") |  |
|  | BMJ Quality & Safety | 6 |
|  | Social Science and Medicine | 4 |
|  | Medical Anthropology | 1 |
|  | Cochrane library | 4 |
|  | Sociology of Health and Illness | 1 |
|  | Implementation Science | 6 |
