# Supplementary material for: Ethnographic research as an evolving method for supporting healthcare improvement skills: a scoping review
Source: BMC Med Res Methodol. 2021 Dec 5;21:274. doi: 10.1186/s12874-021-01466-9 (PMC8647364; doi:10.1186/s12874-021-01466-9)
Supplement: Supplementary file 3 — Additional file 3. [file 12874_2021_1466_MOESM3_ESM.docx]

**Additional File 3: Frequency of most common journals in review**

| Social Science & Medicine | 14 |
| --- | --- |
| Sociology of Health & Illness | 10 |
| Qualitative health research | 9 |
| BMC health services research | 8 |
| Dissertation Abstracts International Section A: Humanities and Social Sciences | 7 |
| Implementation Science | 7 |
| Journal of Clinical Nursing | 7 |
| Social science & medicine (1982) | 7 |
| Journal of Advanced Nursing | 6 |
| Studies in health technology and informatics | 6 |
| Global public health | 5 |
| BMJ open | 4 |
| BMJ quality & safety | 4 |
| Culture, Medicine and Psychiatry | 4 |
| Dissertation Abstracts International: Section B: The Sciences and Engineering | 4 |
| Health | 4 |
| International journal of nursing studies | 4 |
| Journal of Interprofessional Care | 4 |
| Nursing inquiry | 4 |
| Advances in health sciences education : theory and practice | 3 |
| Health policy and planning | 3 |
| International journal of medical informatics | 3 |
| International journal of qualitative studies on health and well-being | 3 |
| Palliative & supportive care | 3 |
| Sociology | 3 |
