# Supplementary material for: Ethnographic research as an evolving method for supporting healthcare improvement skills: a scoping review
Source: BMC Med Res Methodol. 2021 Dec 5;21:274. doi: 10.1186/s12874-021-01466-9 (PMC8647364; doi:10.1186/s12874-021-01466-9)
Supplement: Supplementary file 4 — Additional file 4. [file 12874_2021_1466_MOESM4_ESM.docx]

**Additional File 4: Complete reference list for review**

1. Abdulrehman MS. Reflections on Native Ethnography by a Nurse Researcher. Journal of transcultural nursing : official journal of the Transcultural Nursing Society 2017;28(2):152-58. doi: https://dx.doi.org/10.1177/1043659615620658

2. Aberese-Ako M, van Dijk H, Gerrits T, et al. 'Your health our concern, our health whose concern?': perceptions of injustice in organizational relationships and processes and frontline health worker motivation in Ghana. Health policy and planning 2014;29 Suppl 2:ii15-28. doi: https://dx.doi.org/10.1093/heapol/czu068

3. Ackatia-Armah NM, Addy NA, Ghosh S, et al. Fostering reflective trust between mothers and community health nurses to improve the effectiveness of health and nutrition efforts: An ethnographic study in Ghana, West Africa. Social Science & Medicine 2016;158:96-104. doi: http://dx.doi.org/10.1016/j.socscimed.2016.03.038

4. Adams LV, Basu D, Grande SW, et al. Barriers to tuberculosis care delivery among miners and their families in South Africa: an ethnographic study. The international journal of tuberculosis and lung disease : the official journal of the International Union against Tuberculosis and Lung Disease 2017;21(5):571-78. doi: https://dx.doi.org/10.5588/ijtld.16.0669

5. Adams M, Robert G, Maben J. Exploring the Legacies of Filmed Patient Narratives: The Interpretation and Appropriation of Patient Films by Health Care Staff. Qualitative health research 2015;25(9):1241-50. doi: https://dx.doi.org/10.1177/1049732314566329

6. Adhikari R. Vacant hospitals and under-employed nurses: a qualitative study of the nursing workforce management situation in Nepal. Health policy and planning 2015;30(3):289-97. doi: https://dx.doi.org/10.1093/heapol/czu009

7. Alderson SL, Russell AM, McLintock K, et al. Incentivised case finding for depression in patients with chronic heart disease and diabetes in primary care: an ethnographic study. BMJ Open 2014;4(8):e005146. doi: 10.1136/bmjopen-2014-005146

8. Allard J, Bleakley A. What would you ideally do if there were no targets? An ethnographic study of the unintended consequences of top-down governance in two clinical settings. Advances in Health Sciences Education 2016;21(4):803-17. doi: http://dx.doi.org/10.1007/s10459-016-9667-8

9. Allen D. Lost in translation? 'Evidence' and the articulation of institutional logics in integrated care pathways: from positive to negative boundary object? Sociol Health Illn 2014;36(6):807-22. doi: 10.1111/1467-9566.12111 [published Online First: 2014/03/19]

10. Allen D. Inside 'bed management': Ethnographic insights from the vantage point of UK hospital nurses. Sociology of Health & Illness 2015;37(3):370-84. doi: http://dx.doi.org/10.1111/1467-9566.12195

11. Andersen RS, Vedsted P. Juggling efficiency. An ethnographic study exploring healthcare seeking practices and institutional logics in Danish primary care settings. Social Science & Medicine 2015;128:239-45. doi: http://dx.doi.org/10.1016/j.socscimed.2015.01.037

12. Archer BE. Facilitated conversation groups for people With aphasia: A cognitive ethnographic study. Dissertation Abstracts International: Section B: The Sciences and Engineering 2017;78(4-B(E)):No-Specified.

13. Armstrong N, Brewster L, Tarrant C, et al. Taking the heat or taking the temperature? A qualitative study of a large-scale exercise in seeking to measure for improvement, not blame. Soc Sci Med 2018;198:157-64. doi: 10.1016/j.socscimed.2017.12.033 [published Online First: 2018/01/22]

14. Armstrong N, Herbert G, Brewster L. Contextual barriers to implementation in primary care: An ethnographic study of a programme to improve chronic kidney disease care. Family Practice 2016;33(4):426-31. doi: http://dx.doi.org/10.1093/fampra/cmw049

15. Asan O, Chiou E, Montague E. Quantitative ethnographic study of physician workflow and interactions with electronic health record systems. International Journal of Industrial Ergonomics 2015;49:124-30. doi: http://dx.doi.org/10.1016/j.ergon.2014.04.004

16. Ash JS, Chase D, Wiesen JF, et al. Studying Readiness for Clinical Decision Support for Worker Health Using the Rapid Assessment Process and Mixed Methods Interviews. AMIA Annual Symposium proceedings AMIA Symposium 2016;2016:285-94.

17. Aveling E-L, Parker M, Dixon-Woods M. What is the role of individual accountability in patient safety? A multi-site ethnographic study. Sociology of health & illness 2016;38(2):216-32. doi: https://dx.doi.org/10.1111/1467-9566.12370

18. Aziato L, Adejumo O. The Ghanaian surgical nurse and postoperative pain management: a clinical ethnographic insight. Pain management nursing : official journal of the American Society of Pain Management Nurses 2014;15(1):265-72. doi: https://dx.doi.org/10.1016/j.pmn.2012.10.002

19. Babaei S, Taleghani F, Kayvanara M. Compassionate behaviours of clinical nurses in Iran: An ethnographic study. International Nursing Review 2016;63(3):388-94. doi: http://dx.doi.org/10.1111/inr.12296

20. Baillie J, Lankshear A. Patient and family perspectives on peritoneal dialysis at home: Findings from an ethnographic study. Journal of Clinical Nursing 2015;24(1-2):222-34. doi: http://dx.doi.org/10.1111/jocn.12663

21. Balilla VS, McHenry JA, McHenry MP, et al. The assimilation of Western medicine into a semi-nomadic healthcare system: a case study of the Indigenous Aeta Magbukun, Philippines. EcoHealth 2014;11(3):372-82. doi: https://dx.doi.org/10.1007/s10393-014-0919-x

22. Balka E, Tolar M, Coates S, et al. Socio-technical issues and challenges in implementing safe patient handovers: insights from ethnographic case studies. International journal of medical informatics 2013;82(12):e345-57. doi: https://dx.doi.org/10.1016/j.ijmedinf.2012.11.001

23. Bamford C, Poole M, Brittain K, et al. Understanding the challenges to implementing case management for people with dementia in primary care in England: a qualitative study using Normalization Process Theory. BMC health services research 2014;14:549. doi: https://dx.doi.org/10.1186/s12913-014-0549-6

24. Barreto MdS, Marcon SS, Garcia-Vivar C. Patterns of behaviour in families of critically ill patients in the emergency room: A focused ethnography. Journal of Advanced Nursing 2017;73(3):633-42. doi: http://dx.doi.org/10.1111/jan.13156

25. Batch M, Windsor C. Nursing casualization and communication: a critical ethnography. Journal of advanced nursing 2015;71(4):870-80. doi: https://dx.doi.org/10.1111/jan.12557

26. Belanger E, Rodriguez C, Groleau D, et al. Patient participation in palliative care decisions: An ethnographic discourse analysis. International journal of qualitative studies on health and well-being 2016;11:32438. doi: https://dx.doi.org/10.3402/qhw.v11.32438

27. Benoot C, Bilsen J. An auto-ethnographic study of the disembodied experience of a novice researcher doing qualitative cancer research. Qualitative Health Research 2016;26(4):482-89. doi: http://dx.doi.org/10.1177/1049732315616625

28. Bergenholtz H, Jarlbaek L, Holge-Hazelton B. The culture of general palliative nursing care in medical departments: an ethnographic study. International journal of palliative nursing 2015;21(4):193-201. doi: https://dx.doi.org/10.12968/ijpn.2015.21.4.193

29. Bikker AP, Atherton H, Brant H, et al. Conducting a team-based multi-sited focused ethnography in primary care. BMC Medical Research Methodology 2017;17(1):139. doi: 10.1186/s12874-017-0422-5

30. Bjornsdottir K. The place of standardisation in home care practice: An ethnographic study. Journal of Clinical Nursing 2014;23(9-10):1411-20. doi: http://dx.doi.org/10.1111/jocn.12412

31. Blandford A, Berndt E, Catchpole K, et al. Strategies for conducting situated studies of technology use in hospitals. Cognition, Technology & Work 2015;17(4):489-502. doi: http://dx.doi.org/10.1007/s10111-014-0318-7

32. Boonen MJ, Vosman FJ, Niemeijer AR. Is technology the best medicine? Three practice theoretical perspectives on medication administration technologies in nursing. Nursing inquiry 2016;23(2):121-7. doi: https://dx.doi.org/10.1111/nin.12119

33. Boonen MJMH, Vosman FJH, Niemeijer AR. Tinker, tailor, deliberate. An ethnographic inquiry into the institutionalized practice of bar-coded medication administration technology by nurses. Applied nursing research : ANR 2017;33:30-35. doi: https://dx.doi.org/10.1016/j.apnr.2016.10.007

34. Borrott N, Kinney S, Newall F, et al. Medication communication between nurses and doctors for paediatric acute care: An ethnographic study. Journal of clinical nursing 2017;26(13-14):1978-92. doi: https://dx.doi.org/10.1111/jocn.13606

35. Borycki EM, Kushniruk AW. Use of Techno-Anthropologic Approaches in Studying Technology--induced Errors. Studies in health technology and informatics 2015;215:129-41.

36. Botin L, Bertelsen P, Nohr C. Challenges in Improving Health Care by Use of Health Informatics Technology. Studies in health technology and informatics 2015;215:3-13.

37. Braaf S, Manias E, Riley R. The 'time-out' procedure: an institutional ethnography of how it is conducted in actual clinical practice. BMJ quality & safety 2013;22(8):647-55. doi: https://dx.doi.org/10.1136/bmjqs-2012-001702

38. Brage E, Vindrola-Padros C. An ethnographic exploration of the delivery of psychosocial care to children with cancer in Argentina. European journal of oncology nursing : the official journal of European Oncology Nursing Society 2017;29:91-97. doi: https://dx.doi.org/10.1016/j.ejon.2017.05.002

39. Brenman NF, Hiddinga A, Wright B. Intersecting Cultures in Deaf Mental Health: An Ethnographic Study of NHS Professionals Diagnosing Autism in D/deaf Children. Culture, Medicine, and Psychiatry 2017;41(3):431-52. doi: 10.1007/s11013-017-9526-y

40. Brewster L, Tarrant C, Willars J, et al. Measurement of harms in community care: a qualitative study of use of the NHS Safety Thermometer. BMJ Qual Saf 2017 doi: 10.1136/bmjqs-2017-006970 [published Online First: 2017/12/05]

41. Broer T, Nieboer AP, Bal R. Mutual powerlessness in client participation practices in mental health care. Health expectations : an international journal of public participation in health care and health policy 2014;17(2):208-19. doi: https://dx.doi.org/10.1111/j.1369-7625.2011.00748.x

42. Browne AJ, Varcoe C, Lavoie J, et al. Enhancing health care equity with Indigenous populations: evidence-based strategies from an ethnographic study. BMC Health Services Research 2016;16(1):544. doi: 10.1186/s12913-016-1707-9

43. Brummell SP, Seymour J, Higginbottom G. Cardiopulmonary resuscitation decisions in the emergency department: An ethnography of tacit knowledge in practice. Social Science & Medicine 2016;156:47-54. doi: http://dx.doi.org/10.1016/j.socscimed.2016.03.022

44. Bunce AE, Gold R, Davis JV, et al. Ethnographic process evaluation in primary care: explaining the complexity of implementation. BMC health services research 2014;14:607. doi: https://dx.doi.org/10.1186/s12913-014-0607-0

45. Cable-Williams B, Wilson DM. Dying and death within the culture of long-term care facilities in Canada. International Journal of Older People Nursing 2017;12(1):No-Specified.

46. Campbell ML, Rankin JM. Nurses and electronic health records in a Canadian hospital: examining the social organisation and programmed use of digitised nursing knowledge. Sociology of health & illness 2017;39(3):365-79. doi: https://dx.doi.org/10.1111/1467-9566.12489

47. Caspar S, Ratner PA, Phinney A, et al. The influence of organizational systems on information exchange in long-term care facilities: An institutional ethnography. Qualitative Health Research 2016;26(7):951-65. doi: http://dx.doi.org/10.1177/1049732315619893

48. Chatchumni M, Namvongprom A, Eriksson H, et al. Treating without Seeing: Pain Management Practice in a Thai Context. Pain research & management 2016;2016:9580626. doi: https://dx.doi.org/10.1155/2016/9580626

49. Chretien KC, Tuck MG, Simon M, et al. A Digital Ethnography of Medical Students who Use Twitter for Professional Development. Journal of general internal medicine 2015;30(11):1673-80. doi: https://dx.doi.org/10.1007/s11606-015-3345-z

50. Christensen LR. On intertext in chemotherapy: An ethnography of text in medical practice. Computer Supported Cooperative Work (CSCW) 2016;25(1):1-38. doi: http://dx.doi.org/10.1007/s10606-015-9238-1

51. Collet JP, Skippen PW, Mosavianpour MK, et al. Engaging pediatric intensive care unit (PICU) clinical staff to lead practice improvement: the PICU participatory action research project (PICU-PAR). Implement Sci 2014;9:6. doi: 10.1186/1748-5908-9-6 [published Online First: 2014/01/10]

52. Collier A, Sorensen R, Iedema R. Patients' and families' perspectives of patient safety at the end of life: a video-reflexive ethnography study. International journal for quality in health care : journal of the International Society for Quality in Health Care 2016;28(1):66-73. doi: https://dx.doi.org/10.1093/intqhc/mzv095

53. Collier A, Wyer M. Researching Reflexively With Patients and Families: Two Studies Using Video-Reflexive Ethnography to Collaborate With Patients and Families in Patient Safety Research. Qualitative health research 2016;26(7):979-93. doi: https://dx.doi.org/10.1177/1049732315618937

54. Colmenares-Roa T, Huerta-Sil G, Infante-Castaneda C, et al. Doctor-patient relationship between individuals with fibromyalgia and rheumatologists in public and private health care in Mexico. Qualitative Health Research 2016;26(12):1674-88. doi: http://dx.doi.org/10.1177/1049732315588742

55. Conn LG, Haas B, Cuthbertson BH, et al. Communication and Culture in the Surgical Intensive Care Unit: Boundary Production and the Improvement of Patient Care. Qualitative health research 2016;26(7):895-906. doi: https://dx.doi.org/10.1177/1049732315609901

56. Cooper J, Kierans C. Organ donation, ethnicity and the negotiation of death: Ethnographic insights from the UK. Mortality 2016;21(1):1-18. doi: http://dx.doi.org/10.1080/13576275.2015.1021314

57. Creswell LM. A critical Black feminist ethnography of treatment for women with co-occurring disorders in the psychiatric hospital. The Journal of Behavioral Health Services & Research 2014;41(2):167-84. doi: http://dx.doi.org/10.1007/s11414-013-9344-0

58. Cummings JAF. Pediatric procedural pain: how far have we come? An ethnographic account. Pain management nursing : official journal of the American Society of Pain Management Nurses 2015;16(3):233-41. doi: https://dx.doi.org/10.1016/j.pmn.2014.06.006

59. d'Alessandro E. Human activities and microbial geographies. An anthropological approach to the risk of infections in West African hospitals. Social Science & Medicine 2015;136-137:64-72. doi: http://dx.doi.org/10.1016/j.socscimed.2015.05.016

60. DeKeyser Ganz F, Engelberg R, Torres N, et al. Development of a Model of Interprofessional Shared Clinical Decision Making in the ICU: A Mixed-Methods Study. Critical care medicine 2016;44(4):680-9. doi: https://dx.doi.org/10.1097/CCM.0000000000001467

61. DiCicco-Bloom B, DiCicco-Bloom B. The benefits of respectful interactions: fluid alliancing and inter-occupational information sharing in primary care. Sociology of health & illness 2016;38(6):965-79. doi: https://dx.doi.org/10.1111/1467-9566.12418

62. Dixon-Woods M, Leslie M, Tarrant C, et al. Explaining Matching Michigan: an ethnographic study of a patient safety program. Implementation science : IS 2013;8:70. doi: https://dx.doi.org/10.1186/1748-5908-8-70

63. Dixon-Woods M, Redwood S, Leslie M, et al. Improving quality and safety of care using "technovigilance": an ethnographic case study of secondary use of data from an electronic prescribing and decision support system. The Milbank quarterly 2013;91(3):424-54. doi: https://dx.doi.org/10.1111/1468-0009.12021

64. Doran E, Fleming J, Jordens C, et al. Part of the fabric and mostly right: an ethnography of ethics in clinical practice. The Medical journal of Australia 2015;202(11):587-90.

65. Dossa P. Entangled emplacement: Ethnographic reading of Canadian Muslims' engagement with the world of palliative care. Journal of Muslim Mental Health 2017;11(1):19-38. doi: http://dx.doi.org/10.3998/jmmh.10381607.0011.102

66. Dumbleton S. Goodies and baddies: Equivocal thoughts about families using an autoethnographic approach to explore some tensions between service providers and families of people with learning disabilities. Ethics and Social Welfare 2013;7(3):282-92. doi: http://dx.doi.org/10.1080/17496535.2013.815789

67. Edberg M, Cleary S, Simmons LB, et al. Defining the "community": Applying ethnographic methods for a Latino immigrant health intervention. Human Organization 2015;74(1):27-41. doi: http://dx.doi.org/10.17730/humo.74.1.6561p4u727582850

68. Elder NC, Jacobson CJ, Bolon SK, et al. Patterns of relating between physicians and medical assistants in small family medicine offices. Annals of Family Medicine 2014;12(2):150-57. doi: http://dx.doi.org/10.1370/afm.1581

69. Ewertsson M, Bagga-Gupta S, Blomberg K. Nursing students' socialisation into practical skills. Nurse education in practice 2017;27:157-64. doi: https://dx.doi.org/10.1016/j.nepr.2017.09.004

70. Fleming DJ. Beyond clinical: The exploration and integration of human connection skills in five residency programs at the university of Arizona. Dissertation Abstracts International Section A: Humanities and Social Sciences 2015;76(3-A(E)):No-Specified.

71. Gamlen E, Arber A. First assessments by specialist cancer nurses in the community: an ethnography. European journal of oncology nursing : the official journal of European Oncology Nursing Society 2013;17(6):797-801. doi: https://dx.doi.org/10.1016/j.ejon.2013.03.006

72. Garcia J, Colson PW, Parker C, et al. Passing the baton: Community-based ethnography to design a randomized clinical trial on the effectiveness of oral pre-exposure prophylaxis for HIV prevention among Black men who have sex with men. Contemporary Clinical Trials 2015;45:244-51. doi: https://doi.org/10.1016/j.cct.2015.10.005

73. Gaudet CA. Electronic documentation and nurse-patient interaction. Advances in Nursing Science 2016;39(1):3-14. doi: http://dx.doi.org/10.1097/ANS.0000000000000098

74. Gealogo GA. "A light in the dark": Development of a conceptual model for person-engaged dementia care. Dissertation Abstracts International: Section B: The Sciences and Engineering 2017;77(8-B(E)):No-Specified.

75. Georgiadis A, Corrigan O, Speed E. Frontline healthcare staffs' experience of organizing complex hospital discharges: An ethnographic study. Ethics & Behavior 2017;27(4):335-50. doi: http://dx.doi.org/10.1080/10508422.2016.1200977

76. Gerrish K, Naisby A, Ismail M. Experiences of the diagnosis and management of tuberculosis: a focused ethnography of Somali patients and healthcare professionals in the UK. Journal of advanced nursing 2013;69(10):2285-94. doi: https://dx.doi.org/10.1111/jan.12112

77. Gerrits T. The ambiguity of patient-centred practices: The case of a Dutch fertility clinic. Special Issue: Mediating medical technologies: Flows, frictions and new socialities 2014;21(2):125-35. doi: http://dx.doi.org/10.1080/13648470.2014.914804

78. Gerrits T, Reis R, Braat DDM, et al. Bioethics in practice: Addressing ethically sensitive requests in a Dutch fertility clinic. Social science & medicine (1982) 2013;98:330-9. doi: https://dx.doi.org/10.1016/j.socscimed.2012.12.031

79. Gesbeck MM. Negotiating diabetes: Professional diabetes care work in the U.S. Dissertation Abstracts International Section A: Humanities and Social Sciences 2016;77(1-A(E)):No-Specified.

80. Gillespie A, Moore H. Translating and transforming care: People with brain injury and caregivers filling in a disability claim form. Qualitative Health Research 2016;26(4):532-44. doi: http://dx.doi.org/10.1177/1049732315575316

81. Gillespie BM, Gwinner K, Chaboyer W, et al. Team communications in surgery-Creating a culture of safety. Journal of Interprofessional Care 2013;27(5):387-93. doi: http://dx.doi.org/10.3109/13561820.2013.784243

82. Gkeredakis E, Nicolini D, Swan J. Moral judgments as organizational accomplishments: Insights from a focused ethnography in the English healthcare sector. Language and communication at work: Discourse, narrativity, and organizing 2014:293-324. doi: http://dx.doi.org/10.1093/acprof:oso/9780198703082.003.0012

83. Goldman J, Reeves S, Wu R, et al. Medical residents and interprofessional interactions in discharge: An ethnographic exploration of factors that affect negotiation. Journal of General Internal Medicine 2015;30(10):1454-60. doi: http://dx.doi.org/10.1007/s11606-015-3306-6

84. Goldman J, Reeves S, Wu R, et al. A sociological exploration of the tensions related to interprofessional collaboration in acute-care discharge planning. Journal of interprofessional care 2016;30(2):217-25. doi: https://dx.doi.org/10.3109/13561820.2015.1072803

85. Gordon L, Rees C, Ker J, et al. Using video-reflexive ethnography to capture the complexity of leadership enactment in the healthcare workplace. Advances in health sciences education : theory and practice 2017;22(5):1101-21. doi: https://dx.doi.org/10.1007/s10459-016-9744-z

86. Grant A, Sullivan F, Dowell J. An ethnographic exploration of influences on prescribing in general practice: why is there variation in prescribing practices? Implementation science : IS 2013;8:72. doi: https://dx.doi.org/10.1186/1748-5908-8-72

87. Grant S, Checkland K, Bowie P, et al. The role of informal dimensions of safety in high-volume organisational routines: an ethnographic study of test results handling in UK general practice. Implementation science : IS 2017;12(1):56. doi: https://dx.doi.org/10.1186/s13012-017-0586-8

88. Grant S, Guthrie B. Efficiency and thoroughness trade-offs in high-volume organisational routines: an ethnographic study of prescribing safety in primary care. BMJ Quality &amp; Safety 2018;27(3):199-206. doi: 10.1136/bmjqs-2017-006917

89. Grant S, Mesman J, Guthrie B. Spatio-temporal elements of articulation work in the achievement of repeat prescribing safety in UK general practice. Sociology of Health & Illness 2016;38(2):306-24. doi: http://dx.doi.org/10.1111/1467-9566.12308

90. Griffin CWM. It's a birth not a procedure: An ethnographic study of intrauterine fetal death in a labor and delivery unit of an American hospital setting. Dissertation Abstracts International Section A: Humanities and Social Sciences 2013;73(8-A(E)):No-Specified.

91. Guerra-Reyes L. Implementing a culturally appropriate birthing policy: Ethnographic analysis of the experiences of skilled birth attendants in Peru. Journal of Public Health Policy 2016;37(3):353-68. doi: http://dx.doi.org/10.1057/jphp.2016.19

92. Hagg-Martinell A, Hult H, Henriksson P, et al. Community of practice and student interaction at an acute medical ward: An ethnographic study. Medical teacher 2016;38(8):793-800. doi: https://dx.doi.org/10.3109/0142159X.2015.1104411

93. Hales C, Coombs M, de Vries K. The challenges in caring for morbidly obese patients in Intensive Care: A focused ethnographic study. Australian Critical Care 2018;31(1):37-41. doi: https://doi.org/10.1016/j.aucc.2017.02.070

94. Hales C, de Vries K, Coombs M. Managing social awkwardness when caring for morbidly obese patients in intensive care: A focused ethnography. International journal of nursing studies 2016;58:82-89. doi: https://dx.doi.org/10.1016/j.ijnurstu.2016.03.016

95. Hardon A, Moyer E. Medical technologies: flows, frictions and new socialities. Anthropology & medicine 2014;21(2):107-12. doi: https://dx.doi.org/10.1080/13648470.2014.924300

96. Hilario AP. The stigma experienced by terminally ill patients: Evidence from a Portuguese ethnographic study. Journal of Social Work in End-of-Life & Palliative Care 2016;12(4):331-47. doi: http://dx.doi.org/10.1080/15524256.2016.1247770

97. Hjelm M, Holst G, Willman A, et al. The work of case managers as experienced by older persons (75+) with multi-morbidity - a focused ethnography. BMC geriatrics 2015;15:168. doi: https://dx.doi.org/10.1186/s12877-015-0172-3

98. Hoare KJ, Buetow S, Mills J, et al. Using an emic and etic ethnographic technique in a grounded theory study of information use by practice nurses in New Zealand. Journal of Research in Nursing 2013;18(8):720-31. doi: http://dx.doi.org/10.1177/1744987111434190

99. Hopkinson SG, Wiegand DL. The culture contributing to interruptions in the nursing work environment: An ethnography. Journal of Clinical Nursing 2017;26(23-24):5093-102. doi: http://dx.doi.org/10.1111/jocn.14052

100. Hughes N. Homelessness, health, and literacy: An institutional ethnographic study of the social organization of health care in Ontario, Canada. Dissertation Abstracts International: Section B: The Sciences and Engineering 2018;78(9-B(E)):No-Specified.

101. Ilott I, Gerrish K, Pownall S, et al. Exploring scale-up, spread, and sustainability: an instrumental case study tracing an innovation to enhance dysphagia care. Implement Sci 2013;8:128. doi: 10.1186/1748-5908-8-128 [published Online First: 2013/10/31]

102. Jacoby SF. The insight and challenge of reflexive practice in an ethnographic study of Black traumatically injured patients in Philadelphia. Nursing Inquiry 2017;24(3):No-Specified. doi: http://dx.doi.org/10.1111/nin.12172

103. Jaffre Y, Suh S. Where the lay and the technical meet: Using an anthropology of interfaces to explain persistent reproductive health disparities in West Africa. Social Science & Medicine 2016;156:175-83. doi: http://dx.doi.org/10.1016/j.socscimed.2016.03.036

104. Jangland E, Teodorsson T, Molander K, et al. Inadequate environment, resources and values lead to missed nursing care: A focused ethnographic study on the surgical ward using the Fundamentals of Care framework. Journal of Clinical Nursing 2018;27(11-12):2311-21. doi: doi:10.1111/jocn.14095

105. Jennings BM, Sandelowski M, Higgins MK. Turning over patient turnover: An ethnographic study of admissions, discharges, and transfers. Research in Nursing & Health 2013;36(6):554-66. doi: http://dx.doi.org/10.1002/nur.21565

106. Jensen S. Clinical Simulation: For what and how can it be used in design and evaluation of health IT. Studies in health technology and informatics 2015;215:217-28.

107. Jobling H. Using ethnography to explore causality in mental health policy and practice. Qualitative Social Work: Research and Practice 2014;13(1):49-68. doi: http://dx.doi.org/10.1177/1473325013504802

108. Johannessen LEF. The narrative (re)production of prestige: how neurosurgeons teach medical students to valorise diseases. Social science & medicine (1982) 2014;120:85-91. doi: https://dx.doi.org/10.1016/j.socscimed.2014.09.013

109. Johnson H, Forbes D, Egan MY, et al. Hip-Fracture Care in Rural Southwestern Ontario: An Ethnographic Study of Patient Transitions and Physiotherapy Handoffs. Physiotherapy Canada 2013;65(3):266-75. doi: 10.3138/ptc.2012-19

110. Johnson M, Magnusson C, Allan H, et al. 'Doing the writing' and 'working in parallel': how 'distal nursing' affects delegation and supervision in the emerging role of the newly qualified nurse. Nurse education today 2015;35(2):e29-33. doi: https://dx.doi.org/10.1016/j.nedt.2014.11.020

111. Johnston MS, Hodge E. 'Dirt, death and danger? I don't recall any adverse reaction ...': Masculinity and the taint management of hospital private security work. Gender, Work and Organization 2014;21(6):546-58. doi: http://dx.doi.org/10.1111/gwao.12054

112. Jones L, Exworthy M. Framing in policy processes: A case study from hospital planning in the National Health Service in England. Social Science & Medicine 2015;124:196-204. doi: http://dx.doi.org/10.1016/j.socscimed.2014.11.046

113. Jones L, Exworthy M, Frosini F. Implementing market-based reforms in the English NHS: Bureaucratic coping strategies and social embeddedness. Health Policy 2013;111(1):52-59. doi: http://dx.doi.org/10.1016/j.healthpol.2013.03.010

114. Kakudji Kyungu A. Exacerbation of vulnerability in a hospital setting in Lubumbashi (Democratic Republic of Congo). Global health promotion 2013;20(1 Suppl):51-6. doi: https://dx.doi.org/10.1177/1757975912462423

115. Kalateh Sadati A, Bagheri Lankarani K, Hemmati S. Patients’ Description of Unexpected Interactions: A Critical Ethnography of the Quality of Doctor-Patient Interactions in One Educational Hospital in Shiraz, Iran. Shiraz E-Med J 2016;17(7-8):e59931. doi: 10.17795/semj38269 [published Online First: 2016-08-01]

116. Kaplan AL, Klein MP, Tan HJ, et al. Use of patient ethnography to support quality improvement in benign prostatic hyperplasia. Healthcare 2014;2(4):263-67. doi: https://doi.org/10.1016/j.hjdsi.2014.10.004

117. Kazimierczak KA, Skea Z. 'I've used the word cancer but it's actually good news': discursive performativity of cancer and the identity of urological cancer services. Sociology of health & illness 2015;37(3):340-54. doi: https://dx.doi.org/10.1111/1467-9566.12192

118. Kent F, Francis-Cracknell A, McDonald R, et al. How do interprofessional student teams interact in a primary care clinic? A qualitative analysis using activity theory. Advances in health sciences education : theory and practice 2016;21(4):749-60. doi: https://dx.doi.org/10.1007/s10459-015-9663-4

119. Kerr A. Body work in assisted conception: exploring public and private settings. Sociology of health & illness 2013;35(3):465-78. doi: https://dx.doi.org/10.1111/j.1467-9566.2012.01502.x

120. Kierans C, Padilla-Altamira C, Garcia-Garcia G, et al. When health systems are barriers to health care: Challenges faced by uninsured Mexican kidney patients. PLoS ONE 2013;8(1) doi: http://dx.doi.org/10.1371/journal.pone.0054380

121. Kitchen CEW, Lewis S, Tiffin PA, et al. A focused ethnography of a Child and Adolescent Mental Health Service: factors relevant to the implementation of a depression trial. Trials 2017;18(1):237. doi: https://dx.doi.org/10.1186/s13063-017-1982-8

122. Knauth DR, Meinerz NE. Reflections on sharing data from healthcare anthropological studies. Ciencia & saude coletiva 2015;20(9):2659-66. doi: https://dx.doi.org/10.1590/1413-81232015209.04672015

123. Koenig CJ, Ho EY, Trupin L, et al. An exploratory typology of provider responses that encourage and discourage conversation about complementary and integrative medicine during routine oncology visits. Patient education and counseling 2015;98(7):857-63. doi: https://dx.doi.org/10.1016/j.pec.2015.02.018

124. Kollerup MG, Curtis T, Schantz Laursen B. Visiting nurses’ posthospital medication management in home health care: an ethnographic study. Scandinavian Journal of Caring Sciences 2018;32(1):222-32. doi: doi:10.1111/scs.12451

125. Kooienga S. Rural Patients' and Primary Care Clinic Staffs' Perceptions of EHR Implementation. Journal of Ambulatory Care Management 2018;41(1):71-79. doi: 10.1097/JAC.0000000000000199

126. Kooienga S, Singh RL. Pharmacy and primary care perspectives on e-prescribing in a rural community: A focused ethnography. Research in social & administrative pharmacy : RSAP 2017;13(4):820-30. doi: https://dx.doi.org/10.1016/j.sapharm.2016.08.002

127. Kushniruk AW, Borycki EM. Video Analysis and Remote Digital Ethnography: Approaches to understanding user perspectives and processes involving healthcare information technology. Studies in health technology and informatics 2015;215:206-16.

128. Lalleman PCB, Smid GAC, Lagerwey MD, et al. Nurse middle managers' dispositions of habitus: A Bourdieusian analysis of supporting role behaviors in Dutch and American hospitals. Advances in Nursing Science 2015;38(3):E1-E16.

129. Lalleman PCB, Smid GAC, Lagerwey MD, et al. Curbing the urge to care: A Bourdieusian analysis of the effect of the caring disposition on nurse middle managers' clinical leadership in patient safety practices. International journal of nursing studies 2016;63:179-88. doi: https://dx.doi.org/10.1016/j.ijnurstu.2016.09.006

130. Lamba AR, Linn K, Fletcher KE. Identifying patient safety problems during team rounds: an ethnographic study. BMJ quality & safety 2014;23(8):667-9. doi: https://dx.doi.org/10.1136/bmjqs-2013-002324

131. LeBaron V, Beck SL, Maurer M, et al. An Ethnographic Study of Barriers to Cancer Pain Management and Opioid Availability in India. The Oncologist 2014;19(5):515-22. doi: 10.1634/theoncologist.2013-0435

132. Lee YH. The meaning of early intervention: A parent's experience and reflection on interactions with professionals using a phenomenological ethnographic approach. International journal of qualitative studies on health and well-being 2015;10:25891. doi: https://dx.doi.org/10.3402/qhw.v10.25891

133. Leslie M, Paradis E, Gropper MA, et al. An Ethnographic Study of Health Information Technology Use in Three Intensive Care Units. Health services research 2017;52(4):1330-48. doi: https://dx.doi.org/10.1111/1475-6773.12466

134. Lin F, Chaboyer W, Wallis M, et al. Factors contributing to the process of intensive care patient discharge: An ethnographic study informed by activity theory. International Journal of Nursing Studies 2013;50(8):1054-66. doi: http://dx.doi.org/10.1016/j.ijnurstu.2012.11.024

135. Liu W, Gerdtz M, Manias E. Challenges and opportunities of undertaking a video ethnographic study to understand medication communication. Journal of clinical nursing 2015;24(23-24):3707-15. doi: https://dx.doi.org/10.1111/jocn.12948

136. Liu W, Manias E, Gerdtz M. Medication communication during ward rounds on medical wards: Power relations and spatial practices. Health (London, England : 1997) 2013;17(2):113-34. doi: https://dx.doi.org/10.1177/1363459312447257

137. Liu W, Manias E, Gerdtz M. Medication communication through documentation in medical wards: knowledge and power relations. Nursing inquiry 2014;21(3):246-58. doi: https://dx.doi.org/10.1111/nin.12043

138. Locock L, Robert G, Boaz A, et al. Using a national archive of patient experience narratives to promote local patient-centered quality improvement: an ethnographic process evaluation of 'accelerated' experience-based co-design. Journal of health services research & policy 2014;19(4):200-7. doi: https://dx.doi.org/10.1177/1355819614531565

139. Lynch S. Social workers in pediatric primary care: communication, gender, and scope of practice. Social work in health care 2014;53(2):115-34. doi: https://dx.doi.org/10.1080/00981389.2013.851141

140. Macdonald A. Delivering breast cancer care in urban India: Heterotopia, hospital ethnography and voluntarism. Health & place 2016;39:226-32. doi: https://dx.doi.org/10.1016/j.healthplace.2016.02.003

141. MacDonnell JA. Enhancing our understanding of emancipatory nursing: a reflection on the use of critical feminist methodologies. ANS Advances in nursing science 2014;37(3):271-80. doi: https://dx.doi.org/10.1097/ANS.0000000000000038

142. MacKichan F, Brangan E, Wye L, et al. Why do patients seek primary medical care in emergency departments? An ethnographic exploration of access to general practice. BMJ open 2017;7(4):e013816. doi: https://dx.doi.org/10.1136/bmjopen-2016-013816

143. Mackintosh N, Sandall J. The social practice of rescue: the safety implications of acute illness trajectories and patient categorisation in medical and maternity settings. Sociology of health & illness 2016;38(2):252-69. doi: https://dx.doi.org/10.1111/1467-9566.12339

144. Maes K, Closser S, Kalofonos I. Listening to community health workers: how ethnographic research can inform positive relationships among community health workers, health institutions, and communities. American journal of public health 2014;104(5):e5-9. doi: https://dx.doi.org/10.2105/AJPH.2014.301907

145. Makaroff KS, Storch J, Pauly B, et al. Searching for ethical leadership in nursing. Nursing ethics 2014;21(6):642-58. doi: https://dx.doi.org/10.1177/0969733013513213

146. Manias E. Communication relating to family members' involvement and understandings about patients' medication management in hospital. Health expectations : an international journal of public participation in health care and health policy 2015;18(5):850-66. doi: https://dx.doi.org/10.1111/hex.12057

147. Marcilly R, Beuscart-Zephir M-C. Application of Human Factors Methods to Design Healthcare Work Systems: Instance of the prevention of Adverse Drug Events. Studies in health technology and informatics 2015;215:229-41.

148. Martins MdGT, Castro O, Pereira PPG. Body, stress and nursing: Ethnography of an Intensive Care and Surgical Center. Estudos de Psicologia 2013;30(4):525-37. doi: http://dx.doi.org/10.1590/S0103-166X2013000400006

149. Mason B, Epiphaniou E, Nanton V, et al. Coordination of care for individuals with advanced progressive conditions: a multi-site ethnographic and serial interview study. The British journal of general practice : the journal of the Royal College of General Practitioners 2013;63(613):e580-8. doi: https://dx.doi.org/10.3399/bjgp13X670714

150. Matinga MN, Annegarn HJ, Clancy JS. Healthcare provider views on the health effects of biomass fuel collection and use in rural Eastern Cape, South Africa: an ethnographic study. Social science & medicine (1982) 2013;97:192-200. doi: https://dx.doi.org/10.1016/j.socscimed.2013.08.015

151. May M. Turning the board blue: America's epiduralized system of birth. a medical ethnography. Dissertation Abstracts International Section A: Humanities and Social Sciences 2015;76(5-A(E)):No-Specified.

152. McCabe J, Holmes D. Nursing, sexual health and youth with disabilities: a critical ethnography. Journal of advanced nursing 2014;70(1):77-86. doi: https://dx.doi.org/10.1111/jan.12167

153. McCallum C, Menezes G, Reis APD. The dilemma of a practice: experiences of abortion in a public maternity hospital in the city of Salvador, Bahia. Historia, ciencias, saude--Manguinhos 2016;23(1):37-56. doi: https://dx.doi.org/10.1590/S0104-59702016000100004

154. McCann L, Granter E, Hyde P, et al. Still blue-collar after all these years? An ethnography of the professionalization of emergency ambulance work. Journal of Management Studies 2013;50(5):750-76. doi: http://dx.doi.org/10.1111/joms.12009

155. McCullough MB, Chou AF, Solomon JL, et al. The interplay of contextual elements in implementation: an ethnographic case study. BMC health services research 2015;15:62. doi: https://dx.doi.org/10.1186/s12913-015-0713-7

156. McMurray J, Hicks E, Johnson H, et al. 'Trying to find information is like hating yourself every day': the collision of electronic information systems in transition with patients in transition. Health informatics journal 2013;19(3):218-32. doi: https://dx.doi.org/10.1177/1460458212467547

157. McNeil R, Small W, Lampkin H, et al. "People knew they could come here to get help": An ethnographic study of assisted injection practices at a peer-run 'unsanctioned' supervised drug consumption room in a Canadian setting. AIDS and Behavior 2014;18(3):473-85. doi: http://dx.doi.org/10.1007/s10461-013-0540-y

158. Meeker MA, Waldrop DP, Seo JY. Examining family meetings at end of life: The model of practice in a hospice inpatient unit. Palliative & supportive care 2015;13(5):1283-91. doi: https://dx.doi.org/10.1017/S1478951514001138

159. Mendenhall E, Yarris K, Kohrt BA. Utilization of standardized mental health assessments in anthropological research: Possibilities and pitfalls. Culture, Medicine and Psychiatry 2016;40(4):726-45. doi: http://dx.doi.org/10.1007/s11013-016-9502-y

160. Mengis J, Hohmann K. Temporal work in coordination: Co-orienting around a fleeting object of concern. Language and communication at work: Discourse, narrativity, and organizing 2014:261-91. doi: http://dx.doi.org/10.1093/acprof:oso/9780198703082.003.0011

161. Merchant S, O'Connor M, Halkett G. Time, space and technology in radiotherapy departments: how do these factors impact on patients' experiences of radiotherapy? European journal of cancer care 2017;26(2) doi: https://dx.doi.org/10.1111/ecc.12354

162. Milne J, Greenfield D, Braithwaite J. An ethnographic investigation of junior doctors' capacities to practice interprofessionally in three teaching hospitals. Journal of interprofessional care 2015;29(4):347-53. doi: https://dx.doi.org/10.3109/13561820.2015.1004039

163. Mishra A. 'Trust and teamwork matter': community health workers' experiences in integrated service delivery in India. Global public health 2014;9(8):960-74. doi: https://dx.doi.org/10.1080/17441692.2014.934877

164. Morrison C, Jones M, Jones R, et al. 'You can't just hit a button': an ethnographic study of strategies to repurpose data from advanced clinical information systems for clinical process improvement. BMC medicine 2013;11:103. doi: https://dx.doi.org/10.1186/1741-7015-11-103

165. Mount-Campbell AF, Rayo MF, O'Brien JJ, et al. Patient-Centered Handovers: Ethnographic Observations of Attending and Resident Physicians. Quality Management in Health Care 2016;25(4):225-30. doi: 10.1097/QMH.0000000000000114

166. Murdoch J. Process evaluation for complex interventions in health services research: analysing context, text trajectories and disruptions. BMC health services research 2016;16(1):407. doi: https://dx.doi.org/10.1186/s12913-016-1651-8

167. Mutchler MG, McKay T, McDavitt B, et al. Using peer ethnography to address health disparities among young urban Black and Latino men who have sex with men. American journal of public health 2013;103(5):849-52. doi: https://dx.doi.org/10.2105/AJPH.2012.300988

168. Mylopoulos M, Farhat W. "I can do better": exploring purposeful improvement in daily clinical work. Advances in health sciences education : theory and practice 2015;20(2):371-83. doi: https://dx.doi.org/10.1007/s10459-014-9533-5

169. Nakrem S. Understanding organizational and cultural premises for quality of care in nursing homes: an ethnographic study. BMC health services research 2015;15(1):508.

170. Nambiar D. India's "tryst" with universal health coverage: Reflections on ethnography in Indian health policymaking. Social Science & Medicine 2013;99:135-42. doi: http://dx.doi.org/10.1016/j.socscimed.2013.08.022

171. Nastasi BK, Schensul JJ, Schensul SL, et al. A model for translating ethnography and theory into culturally constructed clinical practices. Culture, Medicine and Psychiatry 2015;39(1):92-120. doi: http://dx.doi.org/10.1007/s11013-014-9404-9

172. Nathan S, Stephenson N, Braithwaite J. Sidestepping questions of legitimacy: how community representatives manoeuvre to effect change in a health service. Health (London, England : 1997) 2014;18(1):23-40. doi: https://dx.doi.org/10.1177/1363459312473617

173. Nelson MM. NICU Culture of Care for Infants with Neonatal Abstinence Syndrome: A Focused Ethnography. Neonatal network : NN 2016;35(5):287-96. doi: https://dx.doi.org/10.1891/0730-0832.35.5.287

174. Nelson P, Bell AJ, Nathanson L, et al. Ethnographic analysis on the use of the electronic medical record for clinical handoff. Internal and Emergency Medicine 2017;12(8):1265-72. doi: 10.1007/s11739-016-1567-7

175. Newnham EC, McKellar LV, Pincombe JI. Paradox of the institution: findings from a hospital labour ward ethnography. BMC Pregnancy and Childbirth 2017;17(1):2. doi: 10.1186/s12884-016-1193-4

176. Ng'ang'a N, Byrne MW, Ngo TA. In their own words: The experience of professional nurses in a Northern Vietnamese women's hospital. Contemporary Nurse 2014;47(1-2):168-79. doi: http://dx.doi.org/10.1080/10376178.2014.11081918

177. Nichols N, Fridman M, Ramadan K, et al. Investigating the social organization of family health work: An institutional ethnography. Critical Public Health 2016;26(5):554-65. doi: http://dx.doi.org/10.1080/09581596.2015.1119804

178. Nightingale R, Sinha MD, Swallow V. Using focused ethnography in paediatric settings to explore professionals' and parents' attitudes towards expertise in managing chronic kidney disease stage 3-5. BMC health services research 2014;14:403. doi: https://dx.doi.org/10.1186/1472-6963-14-403

179. Nilsson G, Hansson K, Tiberg I, et al. How dislocation and professional anxiety influence readiness for change during the implementation of hospital-based home care for children newly diagnosed with diabetes–an ethnographic analysis of the logic of workplace change. BMC health services research 2018;18(1):61.

180. Nilsson L, Eriksen S, Borg C. The influence of social challenges when implementing information systems in a Swedish health-care organisation. Journal of nursing management 2016;24(6):789-97. doi: https://dx.doi.org/10.1111/jonm.12383

181. Norman AH, Russell AJ, Merli C. The Quality and Outcomes Framework: Body commodification in UK general practice. Soc Sci Med 2016;170:77-86. doi: 10.1016/j.socscimed.2016.10.009 [published Online First: 2016/10/21]

182. Nugus P, Forero R, McCarthy S, et al. The emergency department "carousel": an ethnographically-derived model of the dynamics of patient flow. International emergency nursing 2014;22(1):3-9. doi: https://dx.doi.org/10.1016/j.ienj.2013.01.001

183. O’Meara P, Stirling C, Ruest M, et al. Community paramedicine model of care: an observational, ethnographic case study. BMC Health Services Research 2016;16(1):39. doi: 10.1186/s12913-016-1282-0

184. Oster RT, Bruno G, Montour M, et al. Kikiskawâwasow - prenatal healthcare provider perceptions of effective care for First Nations women: an ethnographic community-based participatory research study. BMC Pregnancy and Childbirth 2016;16(1):216. doi: 10.1186/s12884-016-1013-x

185. Oudshoorn A, Ward-Griffin C, Forchuk C, et al. Client-provider relationships in a community health clinic for people who are experiencing homelessness. Nursing inquiry 2013;20(4):317-28. doi: https://dx.doi.org/10.1111/nin.12007

186. Patel B, Patel A, Jan S, et al. A multifaceted quality improvement intervention for CVD risk management in Australian primary healthcare: a protocol for a process evaluation. Implement Sci 2014;9:187. doi: 10.1186/s13012-014-0187-8 [published Online First: 2014/12/18]

187. Patton SJ, Miller FA, Abrahamyan L, et al. Expanding the clinical role of community pharmacy: A qualitative ethnographic study of medication reviews in Ontario, Canada. Health Policy 2018;122(3):256-62. doi: https://doi.org/10.1016/j.healthpol.2017.10.007

188. Pavlish C, Brown-Saltzman K, Jakel P, et al. The nature of ethical conflicts and the meaning of moral community in oncology practice. Oncology nursing forum 2014;41(2):130-40. doi: https://dx.doi.org/10.1188/14.ONF.130-140

189. Person J, Spiva L, Hart P. The culture of an emergency department: an ethnographic study. International emergency nursing 2013;21(4):222-7. doi: https://dx.doi.org/10.1016/j.ienj.2012.10.001

190. Pesut B, Robinson CA, Bottorff JL. Among neighbors: an ethnographic account of responsibilities in rural palliative care. Palliative & supportive care 2014;12(2):127-38. doi: https://dx.doi.org/10.1017/S1478951512001046

191. Pfadenhauer M, Dukat C. Robot caregiver or robot-supported caregiving? The performative deployment of the social robot PARO in dementia care. International Journal of Social Robotics 2015;7(3):393-406. doi: http://dx.doi.org/10.1007/s12369-015-0284-0

192. Pighini MJ, Goelman H, Buchanan M, et al. Learning from parents' stories about what works in early intervention. International Journal of Psychology 2014;49(4):263-70. doi: http://dx.doi.org/10.1002/ijop.12024

193. Portacolone E, Segal SP, Mezzina R, et al. A tale of two cities: The exploration of the Trieste public psychiatry model in San Francisco. Culture, Medicine and Psychiatry 2015;39(4):680-97. doi: http://dx.doi.org/10.1007/s11013-015-9458-3

194. Price AM. Caring and technology in an intensive care unit: An ethnographic study. Nursing in Critical Care 2013;18(6):278-88. doi: http://dx.doi.org/10.1111/nicc.12032

195. Prince RJ, Otieno P. In the shadowlands of global health: observations from health workers in Kenya. Global public health 2014;9(8):927-45. doi: https://dx.doi.org/10.1080/17441692.2014.941897

196. Quilligan S. Learning clinical communication on ward-rounds: an ethnographic case study. Medical teacher 2015;37(2):168-73. doi: https://dx.doi.org/10.3109/0142159X.2014.947926

197. Rajkomar A, Mayer A, Blandford A. Understanding safety-critical interactions with a home medical device through Distributed Cognition. Journal of biomedical informatics 2015;56:179-94. doi: https://dx.doi.org/10.1016/j.jbi.2015.06.002

198. Rajtar M. Health care reform and Diagnosis Related Groups in Germany: The mediating role of Hospital Liaison Committees for Jehovah's Witnesses. Social science & medicine (1982) 2016;166:57-65. doi: https://dx.doi.org/10.1016/j.socscimed.2016.08.016

199. Ranasinghe P. The humdrum of legality and the ordering of an ethic of care. Law & Society Review 2014;48(4):709-39. doi: http://dx.doi.org/10.1111/lasr.12107

200. Reeves S, McMillan SE, Kachan N, et al. Interprofessional collaboration and family member involvement in intensive care units: emerging themes from a multi-sited ethnography. Journal of interprofessional care 2015;29(3):230-7. doi: https://dx.doi.org/10.3109/13561820.2014.955914

201. Reid L, Kydd A, Slade B. An inquiry into what organised difficult advance care planning conversations in a Scottish residential care home using institutional ethnography. Journal of Research in Nursing 2018;23(2-3):220-36. doi: 10.1177/1744987118756477

202. Renedo A, Marston C. Spaces for citizen involvement in healthcare: An ethnographic study. Sociology 2015;49(3):488-504. doi: http://dx.doi.org/10.1177/0038038514544208

203. Rieder S. Tinkering toward departure: The limits of improvisation in rural Ethiopian biomedical practices. Social science & medicine (1982) 2017;179:1-8. doi: https://dx.doi.org/10.1016/j.socscimed.2017.02.023

204. Riley R, Coghill N, Montgomery A, et al. The provision of NHS health checks in a community setting: an ethnographic account. BMC health services research 2015;15:546. doi: https://dx.doi.org/10.1186/s12913-015-1209-1

205. Ringer A, Holen M. "Hell no, they'll think you're mad as a hatter": Illness discourses and their implications for patients in mental health practice. Health: An Interdisciplinary Journal for the Social Study of Health, Illness and Medicine 2016;20(2):161-75. doi: http://dx.doi.org/10.1177/1363459315574115

206. Rixon S, Braaf S, Williams A, et al. The functions and roles of questioning during nursing handovers in specialty settings: An ethnographic study. Contemporary Nurse 2017;53(2):182-95. doi: http://dx.doi.org/10.1080/10376178.2016.1258316

207. Roalkvam S. Health governance in India: citizenship as situated practice. Global public health 2014;9(8):910-26. doi: https://dx.doi.org/10.1080/17441692.2014.941900

208. Rodriguez G, Angelica-Munoz L, Komura Hoga LA. Cultural experiences of immigrant nurses at two hospitals in Chile. Revista Latino-Americana de Enfermagem 2014;22(2):187-96. doi: http://dx.doi.org/10.1590/0104-1169.2980.2401

209. Rodríguez-García C, Falcó-Pegueroles A. Ethnographic analysis of communication and the deaf community’s rights in the clinical context AU - Rodríguez-Martín, Dolors. Contemporary Nurse 2018;54(2):126-38. doi: 10.1080/10376178.2018.1441731

210. Rooshenas L, Owen-Smith A, Hollingworth W, et al. "I won't call it rationing...": An ethnographic study of healthcare disinvestment in theory and practice. Social Science & Medicine 2015;128:273-81. doi: http://dx.doi.org/10.1016/j.socscimed.2015.01.020

211. Ross C, Rogers C, Duff D. Critical ethnography: An under-used research methodology in neuroscience nursing. Canadian journal of neuroscience nursing 2016;38(1):4-7.

212. Russell J, Greenhalgh T. Being 'rational' and being 'human': How National Health Service rationing decisions are constructed as rational by resource allocation panels. Health: An Interdisciplinary Journal for the Social Study of Health, Illness and Medicine 2014;18(5):441-57. doi: http://dx.doi.org/10.1177/1363459313507586

213. Sadati AK, Lankarani KB, Hemmati S. Patients’ description of unexpected interactions: a critical ethnography of the quality of doctor-patient interactions in one educational hospital in Shiraz, Iran. Shiraz E-Medical Journal 2016;17(7-8)

214. Sagasser MH, Fluit CRMG, van Weel C, et al. How Entrustment Is Informed by Holistic Judgments Across Time in a Family Medicine Residency Program: An Ethnographic Nonparticipant Observational Study. Academic medicine : journal of the Association of American Medical Colleges 2017;92(6):792-99. doi: https://dx.doi.org/10.1097/ACM.0000000000001464

215. Saini G, Sampson EL, Davis S, et al. An ethnographic study of strategies to support discussions with family members on end-of-life care for people with advanced dementia in nursing homes. BMC Palliative Care 2016;15(1):55. doi: 10.1186/s12904-016-0127-2

216. Saleem JJ, Plew WR, Speir RC, et al. Understanding barriers and facilitators to the use of Clinical Information Systems for intensive care units and Anesthesia Record Keeping: A rapid ethnography. International journal of medical informatics 2015;84(7):500-11. doi: https://dx.doi.org/10.1016/j.ijmedinf.2015.03.006

217. Salzmann-Erikson M. Using focused ethnography to explore and describe the process of nurses’ shift reports in a psychiatric intensive care unit. Journal of Clinical Nursing 2018;27(15-16):3104-14. doi: doi:10.1111/jocn.14502

218. Scales K, Bailey S, Middleton J, et al. Power, empowerment, and person-centred care: using ethnography to examine the everyday practice of unregistered dementia care staff. Sociology of health & illness 2017;39(2):227-43. doi: https://dx.doi.org/10.1111/1467-9566.12524

219. Scamell M. The fear factor of risk - clinical governance and midwifery talk and practice in the UK. Midwifery 2016;38:14-20. doi: https://dx.doi.org/10.1016/j.midw.2016.02.010

220. Scamell M, Altaweli R, McCourt C. Sarah's birth. How the medicalisation of childbirth may be shaped in different settings: Vignette from a study of routine intervention in Jeddah, Saudi Arabia. Women and birth : journal of the Australian College of Midwives 2017;30(1):e39-e45. doi: https://dx.doi.org/10.1016/j.wombi.2016.08.002

221. Schober MM, Gerrish K, McDonnell A. Development of a conceptual policy framework for advanced practice nursing: an ethnographic study. Journal of advanced nursing 2016;72(6):1313-24. doi: https://dx.doi.org/10.1111/jan.12915

222. Schwitters A, Lederer P, Zilversmit L, et al. Barriers to health care in rural Mozambique: a rapid ethnographic assessment of planned mobile health clinics for ART. Global health, science and practice 2015;3(1):109-16. doi: https://dx.doi.org/10.9745/GHSP-D-14-00145

223. Scott K, McMahon S, Yumkella F, et al. Navigating multiple options and social relationships in plural health systems: a qualitative study exploring healthcare seeking for sick children in Sierra Leone. Health policy and planning 2014;29(3):292-301. doi: https://dx.doi.org/10.1093/heapol/czt016

224. Selman LE, Daveson BA, Smith M, et al. How empowering is hospital care for older people with advanced disease? Barriers and facilitators from a cross-national ethnography in England, Ireland and the USA. Age and ageing 2017;46(2):300-09. doi: https://dx.doi.org/10.1093/ageing/afw193

225. Sercu C, Ayala RA, Bracke P. How does stigma influence mental health nursing identities? An ethnographic study of the meaning of stigma for nursing role identities in two Belgian Psychiatric Hospitals. International Journal of Nursing Studies 2015;52(1):307-16. doi: http://dx.doi.org/10.1016/j.ijnurstu.2014.07.017

226. Sercu C, Bracke P. Stigma as a structural power in mental health care reform: An ethnographic study among mental health care professionals in Belgium. Archives of Psychiatric Nursing 2016;30(6):710-16. doi: http://dx.doi.org/10.1016/j.apnu.2016.06.001

227. Sercu C, Pattyn E, Bracke P. Exploring identity dynamics in mental help-seeking trajectories: An ethnographic study among inpatient service users of two Belgian psychiatric hospitals. Archives of Psychiatric Nursing 2015;29(2):114-19. doi: http://dx.doi.org/10.1016/j.apnu.2014.11.009

228. Sharp S, McAllister M, Broadbent M. The tension between person centred and task focused care in an acute surgical setting: A critical ethnography. Collegian 2018;25(1):11-17. doi: https://doi.org/10.1016/j.colegn.2017.02.002

229. Shaw JA, Kontos P, Martin W, et al. The institutional logic of integrated care: an ethnography of patient transitions. Journal of Health Organization and Management 2017;31(1):82-95. doi: doi:10.1108/JHOM-06-2016-0123

230. Simmonds R, Glogowska M, McLachlan S, et al. Unplanned admissions and the organisational management of heart failure: a multicentre ethnographic, qualitative study. BMJ open 2015;5(10):e007522. doi: https://dx.doi.org/10.1136/bmjopen-2014-007522

231. Smith SA. Migrant encounters in the clinic: Bureaucratic, biomedical, and community influences on patient interactions with front-line workers. Social Science & Medicine 2016;150:49-56. doi: http://dx.doi.org/10.1016/j.socscimed.2015.12.022

232. Spitzmueller MC. Shifting practices of recovery under community mental health reform: A street-level organizational ethnography. Qualitative Social Work: Research and Practice 2014;13(1):26-48. doi: http://dx.doi.org/10.1177/1473325013507472

233. Spitzmueller MC. Negotiating competing institutional logics at the street level: An ethnography of a community mental health organization. Social Service Review 2016;90(1):35-82. doi: http://dx.doi.org/10.1086/686694

234. Stepurko T, Pavlova M, Levenets O, et al. Informal patient payments in maternity hospitals in Kiev, Ukraine. The International journal of health planning and management 2013;28(2):e169-87. doi: https://dx.doi.org/10.1002/hpm.2155

235. Stevens J, Schmied V, Burns E, et al. Video ethnography during and after caesarean sections: Methodological challenges. Journal of Clinical Nursing 2017;26(13-14):2083-92. doi: http://dx.doi.org/10.1111/jocn.13677

236. Stoopendaal A, Bal R. Conferences, tablecloths and cupboards: how to understand the situatedness of quality improvements in long-term care. Soc Sci Med 2013;78:78-85. doi: 10.1016/j.socscimed.2012.11.037 [published Online First: 2012/12/26]

237. Storeng KT. The GAVI Alliance and the 'Gates approach' to health system strengthening. Global public health 2014;9(8):865-79. doi: https://dx.doi.org/10.1080/17441692.2014.940362

238. Strouse SM, Nickerson CJ. Professional culture brokers: Nursing faculty perceptions of nursing culture and their role in student formation. Nurse education in practice 2016;18:10-5. doi: https://dx.doi.org/10.1016/j.nepr.2016.02.008

239. Subramony A, Hametz PA, Balmer D. Family-centered rounds in theory and practice: an ethnographic case study. Academic pediatrics 2014;14(2):200-6. doi: https://dx.doi.org/10.1016/j.acap.2013.11.003

240. Sutherland N, Ward-Griffin C, McWilliam C, et al. Gendered processes in hospice palliative home care for seniors with cancer and their family caregivers. Qualitative Health Research 2016;26(7):907-20. doi: http://dx.doi.org/10.1177/1049732315609571

241. Sutton E, Dixon-Woods M, Tarrant C. Ethnographic process evaluation of a quality improvement project to improve transitions of care for older people. BMJ open 2016;6(8):e010988. doi: https://dx.doi.org/10.1136/bmjopen-2015-010988

242. Swinglehurst D. Displays of authority in the clinical consultation: A linguistic ethnographic study of the electronic patient record. Social Science & Medicine 2014;118:17-26. doi: http://dx.doi.org/10.1016/j.socscimed.2014.07.045

243. Swinglehurst D, Greenhalgh T. Caring for the patient, caring for the record: an ethnographic study of 'back office' work in upholding quality of care in general practice. BMC health services research 2015;15:177. doi: https://dx.doi.org/10.1186/s12913-015-0774-7

244. Szymczak JE. Seeing risk and allocating responsibility: talk of culture and its consequences on the work of patient safety. Soc Sci Med 2014;120:252-9. doi: 10.1016/j.socscimed.2014.09.023 [published Online First: 2014/12/03]

245. Tarrant C, O'Donnell B, Martin G, et al. A complex endeavour: an ethnographic study of the implementation of the Sepsis Six clinical care bundle. Implementation science : IS 2016;11(1):149.

246. Tarrant C, Sutton E, Angell E, et al. The 'weekend effect' in acute medicine: a protocol for a team-based ethnography of weekend care for medical patients in acute hospital settings. BMJ open 2017;7(4):e016755. doi: https://dx.doi.org/10.1136/bmjopen-2017-016755

247. Taylor J, Sims J, Haines TP. The emergent relevance of care staff decision-making and situation awareness to mobility care in nursing homes: an ethnographic study. Journal of advanced nursing 2014;70(12):2767-78. doi: https://dx.doi.org/10.1111/jan.12425

248. Thomas GM, Latimer J. In/exclusion in the clinic: Down's syndrome, dysmorphology and the ethics of everyday medical work. Sociology 2015;49(5):937-54. doi: http://dx.doi.org/10.1177/0038038515588470

249. Tietbohl CK, Rendle KAS, Halley MC, et al. Implementation of Patient Decision Support Interventions in Primary Care: The Role of Relational Coordination. Medical decision making : an international journal of the Society for Medical Decision Making 2015;35(8):987-98. doi: https://dx.doi.org/10.1177/0272989X15602886

250. Tomnay JE, Bourke L, Fairley CK. Exploring the acceptability of online sexually transmissible infection testing for rural young people in Victoria. The Australian Journal of Rural Health 2014;22(1):40-44. doi: http://dx.doi.org/10.1111/ajr.12077

251. Uhrenfeldt L, Hoybye MT. Lived experiences and challenges of older surgical patients during hospitalization for cancer: An ethnographic fieldwork. International Journal of Qualitative Studies on Health and Well-being 2014;9 doi: http://dx.doi.org/10.3402/qhw.v9.22810

252. Van Keer R-L, Deschepper R, Francke AL, et al. Conflicts between healthcare professionals and families of a multi-ethnic patient population during critical care: an ethnographic study. Critical care (London, England) 2015;19:441. doi: https://dx.doi.org/10.1186/s13054-015-1158-4

253. Varley E. Abandonments, solidarities and logics of care: Hospitals as sites of sectarian conflict in Gilgit-Baltistan. Culture, Medicine and Psychiatry 2016;40(2):159-80. doi: http://dx.doi.org/10.1007/s11013-015-9456-5

254. Vasli P, Dehghan-Nayeri N, Borim-Nezhad L, et al. Dominance of paternalism in family-centered care in the pediatric intensive care unit (PICU): an ethnographic study. Issues in comprehensive pediatric nursing 2015;38(2):118-35. doi: https://dx.doi.org/10.3109/01460862.2015.1035464

255. Voldbjerg SL, Gronkjaer M, Wiechula R, et al. Newly graduated nurses' use of knowledge sources in clinical decision-making: An ethnographic study. Journal of Clinical Nursing 2017;26(9-10):1313-27. doi: http://dx.doi.org/10.1111/jocn.13628

256. Wademan DT, Reynolds LJ. Interrogating concepts of care in the HIV care continuum: Ethnographic insights from the implementation of a "Universal Test and Treat" approach in South Africa. AIDS Care 2016;28(Suppl 3):52-58. doi: http://dx.doi.org/10.1080/09540121.2016.1161164

257. Wanchai A, Armer JM, Stewart BR. Thai nurses' perspectives on the use of complementary and alternative medicine among Thai breast cancer survivors in northern Thailand. International journal of nursing practice 2015;21(2):118-24. doi: https://dx.doi.org/10.1111/ijn.12231

258. Waring J, Currie G, Bishop S. A contingent approach to the organization and management of public-private partnerships: An empirical study of English health care. Public Administration Review 2013;73(2):313-26. doi: http://dx.doi.org/10.1111/puar.12020

259. Waring J, Marshall F, Bishop S. Understanding the occupational and organizational boundaries to safe hospital discharge. Journal of health services research & policy 2015;20(1 Suppl):35-44. doi: https://dx.doi.org/10.1177/1355819614552512

260. Waters N. Towards an institutional counter-cartography of nurses' wound work. Journal of Sociology and Social Welfare 2015;42(2):127-56.

261. Weaver RR. Seeking high reliability in primary care: Leadership, tools, and organization. Health care management review 2015;40(3):183-92. doi: https://dx.doi.org/10.1097/HMR.0000000000000022

262. Weaver SH, Lindgren TG, Cadmus E, et al. Report From the Night Shift: How Administrative Supervisors Achieve Nurse and Patient Safety. Nursing administration quarterly 2017;41(4):328-36. doi: https://dx.doi.org/10.1097/NAQ.0000000000000252

263. Webber M, Reidy H, Ansari D, et al. Enhancing social networks: a qualitative study of health and social care practice in UK mental health services. Health & social care in the community 2015;23(2):180-9. doi: https://dx.doi.org/10.1111/hsc.12135

264. Webster F, Fehlings MG, Rice K, et al. Improving access to emergent spinal care through knowledge translation: an ethnographic study. BMC health services research 2014;14:169. doi: https://dx.doi.org/10.1186/1472-6963-14-169

265. Williams-Reade J, Lamson AL, Knight SM, et al. The clinical, operational, and financial worlds of neonatal palliative care: A focused ethnography. Palliative & supportive care 2015;13(2):179-86. doi: https://dx.doi.org/10.1017/S1478951513000916

266. Wilson B, Harwood L, Oudshoorn A. Moving beyond the "perpetual novice": understanding the experiences of novice hemodialysis nurses and cannulation of the arteriovenous fistula. CANNT journal = Journal ACITN 2013;23(1):11-8.

267. Wilson E, Seymour J. The importance of interdisciplinary communication in the process of anticipatory prescribing. International journal of palliative nursing 2017;23(3):129-35. doi: https://dx.doi.org/10.12968/ijpn.2017.23.3.129

268. Wong M-C, Almond H, Cummings E, et al. Patient Centred Systems: Techno-Anthropological reflections on the challenges of 'meaningfully engaging' patients within health informatics research. Studies in health technology and informatics 2015;215:52-66.

269. Wright A, Sittig DF, Ash JS, et al. Lessons learned from implementing service-oriented clinical decision support at four sites: A qualitative study. International journal of medical informatics 2015;84(11):901-11. doi: https://dx.doi.org/10.1016/j.ijmedinf.2015.08.008

270. Wright DK, Brajtman S, Cragg B, et al. Delirium as letting go: An ethnographic analysis of hospice care and family moral experience. Palliative Medicine 2015;29(10):959-66. doi: http://dx.doi.org/10.1177/0269216315580742

271. Xyrichis A, Lowton K, Rafferty AM. Accomplishing professional jurisdiction in intensive care: An ethnographic study of three units. Social science & medicine (1982) 2017;181:102-11. doi: https://dx.doi.org/10.1016/j.socscimed.2017.03.047

272. Yeowell G. 'Oh my gosh I'm going to have to undress': potential barriers to greater ethnic diversity in the physiotherapy profession in the United Kingdom. Physiotherapy 2013;99(4):323-7. doi: https://dx.doi.org/10.1016/j.physio.2013.03.002

273. Zaman S. Silent saviours: family members in a Bangladeshi hospital. Anthropology & medicine 2013;20(3):278-87. doi: https://dx.doi.org/10.1080/13648470.2013.827426

274. Zango Martin I, Flores Martos JA, Moruno Millares P, et al. Occupational therapy culture seen through the multifocal lens of fieldwork in diverse rural areas. Scandinavian journal of occupational therapy 2015;22(2):82-94. doi: https://dx.doi.org/10.3109/11038128.2014.965197
